# Curcumin Supplementation Mitigates Oxytetracycline-Induced Hematological, Immunological, and Oxidative Stress in Rainbow Trout (*Oncorhynchus mykiss*)

**DOI:** 10.3390/vetsci13090904

**Published:** 2026-09-03

**Authors:** Meryem Sedef Dogru, Serpil Mişe Yonar, Mustafa Dorucu, Cemal Orhan, Muhammet Enis Yonar

**Affiliations:** 1 Department of Physiology, Faculty of Medicine, Firat University, Elazig 23200, Türkiye; ms.dogru@firat.edu.tr; 2 Department of Aquaculture, Faculty of Fisheries, Firat University, Elazig 23200, Türkiye; s_mise@firat.edu.tr (S.M.Y.); mdorucu@firat.edu.tr (M.D.); 3 Department of Animal Nutrition and Nutritional Diseases, Faculty of Veterinary Medicine, Firat University, Elazig 23200, Türkiye; corhan@firat.edu.tr

**Keywords:** curcumin, fish, hematology, immunity, oxidative stress, oxytetracycline

## Abstract

Oxytetracycline (OTC) is a broad-spectrum antibiotic widely used in aquaculture for the treatment of bacterial diseases caused by important fish pathogens, including Aeromonas, Pseudomonas, Vibrio, and Lactococcus spp. Although OTC remains an important therapeutic agent in fish farming, its use may also induce undesirable physiological effects, including hematological disturbances, immunosuppression, and oxidative stress. This study investigated the protective effects of dietary curcumin against OTC-induced physiological alterations in rainbow trout (*Oncorhynchus mykiss*). Fish were fed diets supplemented with 0, 10, 50, or 100 mg kg^−1^ curcumin for eight weeks, followed by exposure to 0, 50, and 75 mg L^−1^ OTC for 48 h. Hematological variables, innate immune responses, and oxidative stress biomarkers were comprehensively evaluated. OTC exposure significantly disrupted physiological homeostasis by reducing red blood cell (RBC) count, hemoglobin (Hb), hematocrit (Ht), mean corpuscular volume (MCV), and mean corpuscular hemoglobin (MCH). Furthermore, white blood cell (WBC) count, nitroblue tetrazolium (NBT) activity, phagocytic activity (PA), phagocytic index (PI), total protein (TP), immunoglobulin M (IgM), bactericidal activity (BA), lysozyme activity (LYZ), and myeloperoxidase (MPO) activity were significantly decreased, indicating marked suppression of both cellular and humoral innate immune responses. Oxidative stress was characterized by increased malondialdehyde (MDA) concentrations together with reduced superoxide dismutase (SOD), catalase (CAT), glutathione peroxidase (GSH-Px), and reduced glutathione (GSH) levels, whereas glutathione S-transferase (GST) activity increased following OTC exposure. Dietary curcumin supplementation generally improved hematological and immune indices and was associated with reduced lipid peroxidation and improved antioxidant status. However, the magnitude and statistical pattern of these responses varied among parameters and tissues, and significant CUR × OTC interactions were not observed for all endpoints. Overall, dietary curcumin supplementation progressively attenuated these alterations, although the response pattern varied among parameters. Increasing dietary curcumin supplementation generally led to greater physiological improvement, although the response varied among endpoints. Notably, at 75 mg/L OTC, supplementation with 100 mg/kg curcumin, compared with 0 mg/kg curcumin, increased RBC count by approximately 14.5%, Ht by 47.8%, WBC count by 32.2%, and NBT activity by 38.9%. In the liver, the same treatment reduced MDA by approximately 14.6% and increased SOD activity and GSH levels by 48.7% and 47.2%, respectively (*p* < 0.001 for all). The highest dietary curcumin level generally produced the greatest physiological improvement. In conclusion, dietary curcumin effectively mitigated OTC-induced hematological, immunological, and oxidative disturbances in rainbow trout, highlighting its considerable potential as a functional feed additive to improve fish health, enhance physiological resilience under antibiotic-associated stress, and support more sustainable aquaculture practices.

## Simple Summary

Oxytetracycline is an antibiotic commonly used to treat bacterial diseases in farmed fish, but its use may also cause unwanted effects on fish health. Curcumin, a natural compound obtained from turmeric, has antioxidant and immune-supporting properties and may help reduce these adverse effects. This study investigated whether adding curcumin to the diet could protect rainbow trout against physiological disturbances caused by oxytetracycline. Fish were fed diets containing different levels of curcumin for eight weeks and were subsequently exposed to different concentrations of oxytetracycline. Oxytetracycline adversely affected blood characteristics, immune responses, and the balance between oxidative damage and antioxidant defenses. Dietary curcumin generally reduced these changes, and higher dietary levels tended to provide greater protection, although the degree of improvement varied among the parameters and tissues examined. The highest tested curcumin level generally produced the most favorable overall response. These findings suggest that dietary curcumin may be a useful natural feed additive for supporting fish health during antibiotic-associated stress. Further studies are needed to determine its long-term effectiveness, safety, and practical application under commercial aquaculture conditions.

## 1. Introduction

The rapid expansion of aquaculture has been accompanied by an increase in infectious and stress-related diseases, especially under intensive production conditions. To maintain fish health and productivity, a wide range of veterinary drugs and chemicals are routinely used in aquaculture systems. These include disinfectants, antiparasitic agents, anesthetics, and therapeutic compounds designed to prevent and control disease outbreaks [1]. The use of these substances has become an essential part of modern aquaculture; however, their extensive and sometimes improper application has raised concerns about environmental contamination, residue buildup, and potential effects on aquatic ecosystems [2]. Among these agents, antibiotics are the most commonly used therapeutic group, primarily targeting bacterial infections that often occur in intensive farming systems. They are usually administered via medicated feed or immersion, depending on the disease type and farming conditions [1,3]. Despite their effectiveness, antibiotic use in aquaculture is often influenced by prophylactic practices, limited regulatory oversight, and inconsistent management strategies, which can lead to overuse or misuse [4]. The widespread use of antibiotics has raised concerns about antimicrobial resistance, environmental persistence, and unintended physiological effects in fish. In addition to contributing to the development of resistance, some antibiotics can induce stress responses, including changes in hematological profiles, modulation of immune function, and disruption of oxidative balance. These effects can compromise fish health and reduce their ability to handle additional stressors [5].

Following the widespread use of antibiotics in aquaculture, oxytetracycline (OTC) has become one of the most commonly used broad-spectrum antimicrobial agents, especially in intensive farming systems. It is widely applied against bacterial pathogens such as *Aeromonas*, *Pseudomonas*, *Vibrio*, and *Lactococcus* spp., and is administered in many economically important fish species, usually through medicated feed at doses of approximately 75–80 mg/kg of body weight for 7–10 days [6,7]. Despite its effectiveness, OTC has been linked to several adverse effects in fish. Extended or high-dose exposure can result in hepatotoxicity and tissue damage, while even short-term treatments may alter hematological parameters, induce oxidative stress, and disturb gut microbiota balance [8,9]. Additionally, OTC is primarily excreted unchanged and can persist in aquatic environments, leading to sediment buildup and the spread of antibiotic resistance [6,10].

Given the widespread and often intensive use of antibiotics in aquaculture, growing evidence indicates that these agents can cause oxidative stress, disrupt immune balance, and damage fish tissues. These unintended physiological effects not only harm fish health but may also decrease treatment effectiveness in farming conditions. As a result, there is a rising demand for effective strategies to reduce antibiotic-induced toxicity while maintaining their therapeutic benefits. In this context, natural bioactive compounds have emerged as promising functional feed additives because they can boost antioxidant defenses, modulate immune responses, and enhance overall physiological resilience in aquaculture species. Curcumin, the main bioactive compound from *Curcuma longa*, has been extensively studied for its wide range of biological activities, including antioxidant, anti-inflammatory, antimicrobial, and immunomodulatory effects [11]. At the molecular level, curcumin protects by regulating key signaling pathways involved in oxidative stress and inflammation, such as activating antioxidant defenses and suppressing pro-inflammatory mediators [12]. Its ability to neutralize reactive oxygen species and boost natural antioxidant enzymes has been well documented, demonstrating its potential to combat oxidative damage from environmental and chemical stressors [13]. Beyond its antioxidant effects, curcumin also shows significant immunomodulatory and antimicrobial activity, helping improve host defenses and disease resistance. Although it has bioavailability limitations, recent studies highlight its safety and therapeutic potential across many biological systems [14]. Overall, these features suggest that curcumin could be a valuable dietary supplement for reducing drug-induced physiological issues and improving fish health.

Although the antioxidant and immunomodulatory properties of curcumin have been extensively documented under a wide range of environmental and chemical stress conditions, considerably less attention has been paid to its potential to alleviate the physiological disturbances associated with antibiotic exposure in fish. OTC remains one of the most widely used antibiotics in aquaculture; however, information on effective nutritional strategies to mitigate its unintended adverse effects is still limited. Since OTC-induced oxidative stress and immune dysfunction may compromise fish health and reduce the overall effectiveness of therapeutic treatments, identifying natural dietary compounds that can minimize these undesirable side effects has become increasingly important. To the best of our knowledge, studies specifically evaluating the protective effects of dietary curcumin against OTC-induced hematological, immunological, and oxidative stress responses in rainbow trout (*Oncorhynchus mykiss*) remain scarce. Accordingly, investigating curcumin’s potential to counteract OTC-induced physiological disturbances may provide valuable insights into its practical use as a functional feed additive to improve fish health and resilience during antibiotic therapy in aquaculture.

Therefore, the present study aimed to investigate whether dietary curcumin supplementation could mitigate OTC-induced physiological disturbances in rainbow trout. To achieve this aim, hematological, immunological, and oxidative stress responses were evaluated across multiple tissues, allowing assessment of the dose-dependent effects of curcumin and its interaction with OTC exposure. It was hypothesized that curcumin would attenuate OTC-induced alterations by enhancing antioxidant defenses and preserving immune homeostasis. The integrated evaluation of these responses across graded curcumin and OTC levels constitutes the principal novelty of the study.

## 2. Materials and Methods

### 2.1. Ethics Statement

All experimental procedures were conducted in accordance with national and institutional guidelines for the care and use of experimental animals and were approved by the Local Animal Ethics Committee of Firat University (FÜHADYEK, Elazig, Türkiye; 2026/03-02, Date: 3 February 2026). The study protocol was reviewed and authorized prior to the initiation of the experiment.

During the preparation of this manuscript, a generative artificial intelligence tool was used solely for English-language editing to improve the clarity and readability of the text. The tools were not used for research design, experimental procedures, data generation, statistical analysis, or scientific interpretation of the results. The authors reviewed and edited all AI-assisted text.

### 2.2. Chemicals

Curcumin derived from *Curcuma longa* (≥65% purity by HPLC) was obtained from Sigma-Aldrich (catalog No. C1386, St. Louis, MO, USA). OTC (OKSIFISH 75%; containing oxytetracycline hydrochloride equivalent to 750 mg oxytetracycline base per g) was purchased from Medicavet (Istanbul, Türkiye). All other chemicals and reagents used in the biochemical analyses were of analytical grade.

### 2.3. Diet Preparation

The basal diet consisted of a commercial trout grower pellet (Ecobio; Cagatay Oil and Feed Products Inc., Izmir, Türkiye) containing 45% crude protein, 20% crude fat, 11% ash, 3% crude fiber, 8.5% moisture, 12.5% nitrogen-free extract, and 5124 kcal/kg gross energy. As the basal formulation contained no Curcuma longa or other curcumin-containing ingredients, its curcumin level was considered 0 mg/kg. Experimental diets were prepared by thoroughly mixing the finely ground basal feed with curcumin at 0, 10, 50, or 100 mg/kg, followed by pelleting to a diameter of 1 mm using a mechanical pellet machine, as described by Komal et al. [15]. The pellets were air-dried at room temperature, stored at 4–5 °C, and used throughout the 8-week feeding period. The curcumin concentrations in the finished pelleted diets were not analytically verified after feed preparation; therefore, the reported dietary curcumin concentrations represent nominal inclusion levels based on the formulated amounts.

During this period, fish were fed three times daily (09:00, 13:00, and 17:00) to apparent satiation. After each feeding, any uneaten feed was carefully removed from the tanks to preserve water quality. Feed consumption was monitored qualitatively throughout the experimental period; however, quantitative feed intake data were not recorded, and therefore the exact daily curcumin intake per fish could not be calculated.

### 2.4. Experimental Fish and Rearing Conditions

Rainbow trout (*Oncorhynchus mykiss*) with an average body weight of approximately 40 g were obtained from a commercial fish farm and transferred to the Fisheries Research Center of Fırat University. Fish were acclimated for 15 days in 540 L fiberglass tanks under controlled conditions. During acclimation, fish were fed a commercial diet and maintained under standard aquaculture conditions.

Water quality parameters were monitored regularly throughout the experimental period. Partial water exchange was performed daily at approximately 40–50% of the tank volume, while complete water renewal was carried out once per week. Water temperature was maintained at 15 ± 1 °C, dissolved oxygen levels were kept at approximately 7.2 ± 0.4 mg L^−1^, and pH was maintained within the range of 7.4 ± 0.3.

### 2.5. Experimental Design

**Figure 1:**
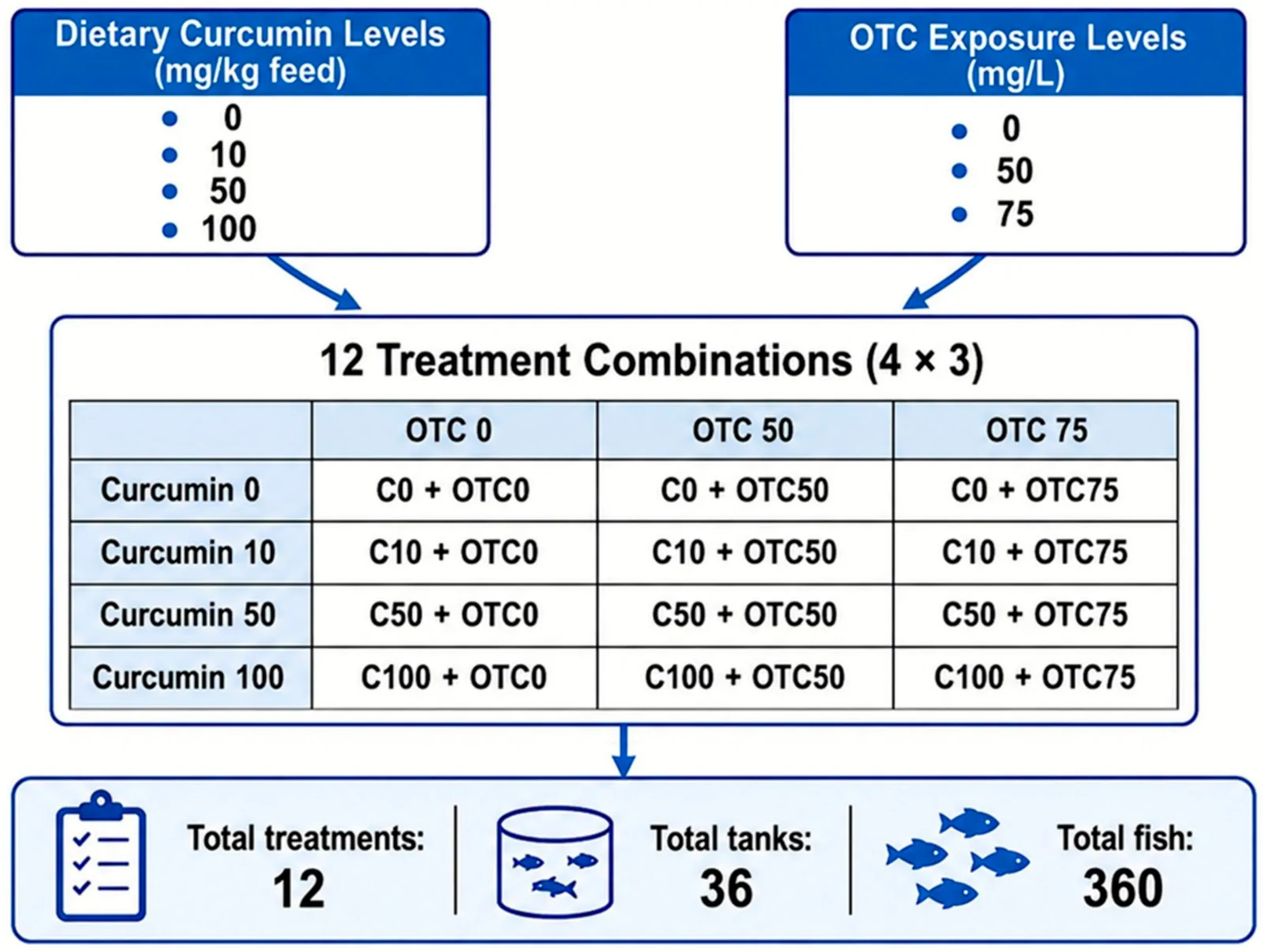
Schematic representation of the experimental design. The study followed a 4 × 3 factorial arrangement comprising four dietary curcumin levels (0, 10, 50, and 100 mg/kg feed) and three oxytetracycline (OTC) exposure levels (0, 50, and 75 mg/L), resulting in 12 treatment combinations (n = 30).

The experimental design followed a completely randomized, factorial arrangement to investigate the individual and combined effects of dietary curcumin and OTC-induced stress in rainbow trout. After a 15-day acclimation period, a total of 360 fish (average body weight: approximately 40 g) were randomly allocated to 36 experimental tanks (10 fish per tank), and the tanks were subsequently randomly assigned to 12 treatment combinations (*n* = 30). Tank positions remained fixed throughout the study, while standardized environmental and husbandry conditions were maintained across all tanks to minimize potential positional effects. The experiment followed a 4 × 3 factorial design consisting of four dietary curcumin levels (0, 10, 50, and 100 mg/kg feed) and three OTC exposure levels (0, 50, and 75 mg/L), resulting in 12 treatment combinations (Figure 1). Fish were fed the assigned curcumin-supplemented diets for 8 weeks, after which they were exposed to the designated OTC concentration by immersion for the final 48 h of the experiment. Immersion exposure was selected to ensure a rapid and relatively uniform OTC challenge while minimizing variability arising from individual feed intake. During the 48 h OTC immersion period, no water renewal was performed, and fish were maintained continuously in the same exposure water. Water temperature, dissolved oxygen, and pH were maintained at approximately 15 ± 1 °C, 7.2 ± 0.4 mg/L, and 7.4 ± 0.3, respectively. The OTC concentrations of 50 and 75 mg/L were nominal exposure concentrations based on the amounts added to the water and were not analytically verified during the 48 h exposure period. Ammonia concentrations were not specifically quantified during the static exposure period. The concentrations of 50 and 75 mg/L were chosen to provide graded sublethal exposure levels based on previously reported experimental immersion protocols in fish [16]. No mortality was observed in any experimental group throughout the study period.

This design enabled the evaluation of the main effects of curcumin supplementation and OTC exposure, as well as their interaction effects on physiological parameters. Additionally, the graded inclusion levels of curcumin allowed assessment of linear and nonlinear dose-related responses.

### 2.6. Sample Collection and Preparation

All fish were anesthetized using benzocaine solution, and blood samples were collected from the caudal vein using sterile syringes. A portion of the blood was transferred into tubes containing K_3_EDTA for hematological and immunological analyses. Hematological parameters included red blood cell (RBC) count, hemoglobin (Hb) concentration, hematocrit (Ht), and erythrocyte indices: mean corpuscular volume (MCV), mean corpuscular hemoglobin (MCH), and mean corpuscular hemoglobin concentration (MCHC). Immunological parameters included white blood cell (WBC) count, respiratory burst activity of neutrophils determined by the nitroblue tetrazolium (NBT) assay, as well as phagocytic activity (PA) and phagocytic index (PI).

The remaining blood samples were transferred into serum tubes and allowed to clot at room temperature for approximately 30 min. Serum was then separated by centrifugation at 5000× *g* for 10 min and stored at −20 °C until further analysis. Serum samples were used to determine total protein (TP), immunoglobulin M (IgM), bactericidal activity (BA), lysozyme activity (LYZ), and myeloperoxidase (MPO) activity.

Following blood collection, tissue samples from the liver, kidney, spleen, and gill were carefully excised, rinsed with physiological saline (0.9% NaCl), and stored at −40 °C until biochemical analyses were conducted within 1 month. Tissues were homogenized in ice-cold 1.15% KCl solution at a 1:10 (*w*/*v*) ratio using a homogenizer. The homogenates were centrifuged at 18,000× *g* for 30 min at 4 °C, and the resulting supernatants were used for the determination of malondialdehyde (MDA) levels as an indicator of lipid peroxidation and antioxidant enzyme activities, including superoxide dismutase (SOD), catalase (CAT), glutathione peroxidase (GSH-Px), and glutathione S-transferase (GST). In addition, reduced glutathione (GSH) levels were measured as a marker of non-enzymatic antioxidant status.

### 2.7. Analysis of Hematological Parameters

Red blood cell (RBC) counts were determined with a hemocytometer after appropriate dilution with Natt and Herrick solution. Hemoglobin (Hb) concentration was measured spectrophotometrically using Drabkin’s reagent at 540 nm by the cyanmethemoglobin method. Hematocrit (Ht) was determined by the microhematocrit centrifugation method. Erythrocyte indices, including mean corpuscular volume (MCV), mean corpuscular hemoglobin (MCH), and mean corpuscular hemoglobin concentration (MCHC), were calculated from the measured RBC, Hb, and Ht values using the following equations [17,18]:

MCV (µm^3^) = (Hematocrit × 10)/RBC count

MCH (pg) = (Hemoglobin × 10)/RBC count

MCHC (%) = (Hemoglobin × 100)/Hematocrit

All hematological measurements were conducted under standardized laboratory conditions to ensure analytical consistency and reliability.

### 2.8. Analysis of Immunological Parameters

#### 2.8.1. White Blood Cell Count

WBC counts were determined simultaneously with red blood cell counts using a hemocytometer following appropriate dilution procedures.

#### 2.8.2. Oxidative Radical Production

The production of reactive oxygen species by phagocytes was assessed using the NBT reduction assay. Briefly, 0.1 mL of whole blood was mixed with an equal volume of 0.2% NBT solution in microplate wells and incubated at room temperature for 30 min. After incubation, 0.05 mL of the mixture was transferred into tubes containing 1.0 mL of N, N-dimethylformamide. Following centrifugation, the absorbance of the supernatant was measured at 620 nm [19,20].

#### 2.8.3. Phagocytic Activity and Phagocytic Index

PA and PI were determined using heparinized blood samples. Briefly, 0.1 mL of blood was mixed with 0.1 mL of a bacterial suspension containing approximately 1 × 10^7^ cells of *Yersinia ruckeri* in phosphate-buffered saline (PBS) and incubated for 30 min. After incubation, a small aliquot was smeared onto glass slides, air-dried, fixed in ethanol, and stained with Giemsa solution. A total of 100 phagocytes per slide were examined under a microscope. PA and PI were calculated using the following equations [21]:

PA (%) = (Number of phagocytic cells containing bacteria/Total number of phagocytes) × 100

PI = (Total number of engulfed bacteria/Number of phagocytic cells)

#### 2.8.4. Total Protein Level

Serum TP levels were determined using a modified Biuret method. Serum samples were diluted with distilled water, followed by the addition of Biuret reagent. After incubation at room temperature in the dark, absorbance was measured at 540 nm [19].

#### 2.8.5. Total Immunoglobulin Level

Total IgM levels were estimated based on polyethylene glycol precipitation. Total protein concentrations were measured before and after precipitation, and the difference between these values was considered as the IgM content [19].

#### 2.8.6. Serum Bactericidal Activity

BA was evaluated by mixing equal volumes of serum and bacterial suspension, followed by incubation at 25 °C for 1 h. The mixture was then diluted with sterile phosphate-buffered saline and plated onto tryptic soy agar (TSA). After incubation, the number of viable bacterial colonies was counted [22].

#### 2.8.7. Lysozyme Activity

LYZ activity was determined using a turbidimetric assay based on the lysis of *Micrococcus lysodeikticus*. Serum samples were added to a bacterial suspension prepared in sodium phosphate buffer, and the decrease in absorbance was monitored at 450 nm. One unit of lysozyme activity was defined as a decrease in absorbance of 0.001 per minute [23].

#### 2.8.8. Myeloperoxidase Activity

MPO activity was measured using a colorimetric method. Serum samples were diluted with Hank’s balanced salt solution, followed by the addition of tetramethylbenzidine (TMB) and hydrogen peroxide. The reaction was stopped by adding sulphuric acid, and absorbance was read at 450 nm [24,25].

### 2.9. Analyses of Oxidative Stress and Antioxidant Capacity

#### 2.9.1. Tissue Malondialdehyde Level

Lipid peroxidation in tissue samples was evaluated by determining MDA levels using the thiobarbituric acid reactive substances (TBARS) method [26]. Tissue homogenates were reacted with thiobarbituric acid under acidic conditions and high temperature to form a colored complex. The absorbance of this complex was measured spectrophotometrically, and MDA concentrations were calculated using a standard curve prepared with 1,1,3,3-tetramethoxypropane.

#### 2.9.2. Tissue Superoxide Dismutase Activity

SOD activity was determined based on its ability to inhibit the reduction of NBT by superoxide radicals generated in a xanthine–xanthine oxidase system. The reaction mixture containing xanthine, EDTA, bovine serum albumin, and NBT was combined with the sample, and the reaction was initiated by adding xanthine oxidase. After incubation at 25 °C, the reaction was terminated, and absorbance was measured at 560 nm [27].

#### 2.9.3. Tissue Catalase Activity

CAT activity was measured by monitoring the decomposition rate of hydrogen peroxide (H_2_O_2_). The reaction mixture consisted of phosphate buffer and H_2_O_2_, to which the tissue sample was added. The decrease in absorbance at 240 nm was recorded over time, and enzyme activity was expressed as the rate constant of H_2_O_2_ degradation per mg protein [28].

#### 2.9.4. Tissue Glutathione Peroxidase Activity

GSH-Px activity was determined by measuring the rate of NADPH oxidation at 340 nm [29]. The assay system included phosphate buffer, EDTA, sodium azide, GSH, NADPH, glutathione reductase, and hydrogen peroxide. The decrease in absorbance was monitored spectrophotometrically, and enzyme activity was expressed in terms of NADPH consumption per unit time.

#### 2.9.5. Tissue Glutathione S-Transferase Activity

GST activity was measured spectrophotometrically based on the conjugation of reduced glutathione with a suitable substrate such as 1-chloro-2,4-dinitrobenzene. The increase in absorbance was recorded at 340 nm, and enzyme activity was expressed per mg protein [30].

#### 2.9.6. Reduced Glutathione Level

GSH levels in tissue samples were determined using a colorimetric method based on its reaction with 5,5′-dithiobis-(2-nitrobenzoic acid) (DTNB). The resulting yellow-colored product was measured spectrophotometrically, and GSH concentrations were calculated using a standard curve [31].

#### 2.9.7. Tissue Protein Content

Total protein levels in tissue homogenates were determined using the Lowry method, with bovine serum albumin used as the standard for calibration [32].

### 2.10. Statistical Analysis

The experiment was designed as a completely randomized factorial arrangement including four dietary levels of curcumin (0, 10, 50, and 100 mg/kg feed) and three levels of OTC exposure (0, 50, and 75 mg/L).

All data were expressed as mean and standard deviation. Normality of the data distribution and homogeneity of variances were assessed using the Shapiro–Wilk and Levene’s tests, respectively, and both assumptions were confirmed for the analyzed endpoints.

To determine the main effects of dietary curcumin and OTC exposure, as well as their interaction, a two-way analysis of variance (Two-way ANOVA) was performed using the General Linear Model (GLM) procedure. When significant effects were detected, Tukey’s multiple-comparison test was used for pairwise comparisons, thereby controlling the family-wise error rate within each endpoint. No additional global FDR correction was applied across the predefined biologically distinct endpoints.

Polynomial contrast analysis was performed to evaluate linear, quadratic, and cubic trends in response to increasing dietary curcumin supplementation at each OTC treatment level.

The statistical model was as follows:

*Y_ijk_* = *μ* + *CUR_i_* + *OTC_j_* + (*CUR* × *OTC*)*ij* + *ε_ijk_*,

where *Y* is the observed response variable, *μ* is the overall mean, CUR is the fixed effect of dietary curcumin supplementation, OTC is the fixed effect of OTC treatment, (CUR × OTC) is the interaction between the two fixed factors, and *ε* is the residual error.

All statistical analyses were performed using IBM SPSS Statistics for Windows, Version 22.0 (IBM Corp., Armonk, NY, USA), and graphs were generated using GraphPad Prism version 11.0 (GraphPad Software, San Diego, CA, USA). Differences were considered statistically significant at *p* < 0.05.

## 3. Results

### 3.1. Hematological Responses

OTC treatment markedly altered all hematological variables evaluated in the present study (Table 1). RBC count, Hb concentration, Ht, MCV, and MCH declined progressively with increasing OTC concentration, and the lowest mean values for these variables were consistently recorded in fish exposed to 75 mg/L OTC (main effect, *p* < 0.001). MCHC also differed significantly among OTC treatments, although the magnitude of change was less pronounced than for the other erythrocyte indices.

Among fish receiving no OTC, dietary curcumin supplementation produced a clear dose-related response in erythrocyte profiles (Table 1). RBC, Ht, MCV, and MCH increased progressively with increasing dietary curcumin levels, with the highest values generally observed in fish fed 100 mg/kg curcumin. RBC count plateaued at 50 and 100 mg/kg, whereas MCHC showed only slight fluctuations despite statistically significant differences among dietary treatments. Hb concentration also increased with dietary curcumin supplementation, although differences among the four dietary groups were less distinct than those observed for the remaining erythrocyte-related variables.

When all experimental groups were considered collectively, dietary curcumin supplementation exerted a significant main effect on all hematological variables (Table 1). Overall, increasing dietary curcumin inclusion levels were associated with higher RBC, Hb, Ht, MCV, and MCH values (*p* < 0.001), whereas the response of MCHC was comparatively less pronounced.

Interaction analysis identified significant CUR × OTC effects for RBC, Ht, MCV, MCH, and MCHC, whereas no interaction effect was detected for Hb (*p* = 0.609) (Table 1). Within both OTC-treated groups, increasing dietary curcumin levels were generally associated with higher RBC, Hb, Ht, MCV, and MCH values. However, the CUR × OTC interaction was significant for RBC, Ht, MCV, MCH, and MCHC, but not for Hb. Fish receiving 100 mg/kg dietary curcumin generally exhibited the highest hematological values under OTC exposure, whereas the lowest values were consistently observed in fish exposed to 75 mg/L OTC without dietary curcumin supplementation. Polynomial contrast analysis further demonstrated predominantly linear dose-related responses to dietary curcumin across OTC treatments, whereas significant quadratic and cubic trends were restricted to selected hematological variables (Table 1).

### 3.2. Immune Responses

OTC treatment significantly affected all evaluated immune parameters (Table 2 and Table 3). WBC count, NBT activity, PA, PI, and TP decreased progressively as OTC concentration increased. The lowest mean values for WBC, NBT, PA, and TP were consistently observed in fish exposed to 75 mg/L OTC, whereas PI remained similarly reduced across both OTC-treated groups (main effect: *p* < 0.001 for all variables).

Among fish receiving no OTC treatment, dietary curcumin supplementation produced a distinct graded response in immune parameters (Table 2 and Table 3). WBC count, NBT activity, PA, PI, and TP increased progressively with increasing dietary curcumin levels, with the highest values generally observed in fish fed 100 mg/kg curcumin. The response was more pronounced for WBC count, NBT activity, and TP, whereas PI exhibited comparatively smaller changes among dietary treatments.

When all experimental groups were evaluated collectively, dietary curcumin supplementation exerted a significant main effect on all immune parameters (Table 2 and Table 3). Overall, increasing dietary curcumin inclusion levels were associated with higher WBC count, NBT activity, PA, PI, and TP values (*p* < 0.001 for all variables).

Interaction analysis identified significant CUR × OTC effects for WBC count and NBT activity, whereas no interaction was detected for PA, PI, or TP (Table 2 and Table 3). Within both OTC-treated groups, increasing dietary curcumin levels were generally associated with higher immune parameter values. However, significant CUR × OTC interactions were detected only for selected endpoints, including WBC and NBT, whereas PA, PI, and TP showed significant curcumin main effects without significant interaction. Polynomial contrast analysis further demonstrated predominantly linear dose-related responses to dietary curcumin across OTC treatments, while significant quadratic and cubic trends were observed only for selected immune parameters.

### 3.3. Oxidative Stress and Antioxidant Capacity

#### 3.3.1. Tissue MDA Levels

OTC treatment significantly influenced MDA concentrations in all examined tissues, including the liver, kidney, spleen, and gills (Table 4, Table 5, Table 6 and Table 7). A progressive increase in tissue MDA levels was observed with increasing OTC concentration, and the highest values were consistently detected in fish exposed to 75 mg/L OTC (main effect, *p* < 0.001 for all tissues).

Among fish receiving no OTC treatment, dietary curcumin supplementation produced a clear dose-dependent reduction in MDA concentrations across all examined tissues (Table 4, Table 5, Table 6 and Table 7). The lowest MDA levels were generally observed in fish fed 100 mg/kg curcumin.

When all experimental groups were evaluated collectively, dietary curcumin supplementation exerted a significant main effect on tissue MDA concentrations in all organs examined (Table 4, Table 5, Table 6 and Table 7). Overall, increasing dietary curcumin inclusion levels were associated with progressively lower MDA values in the liver, kidney, spleen, and gills (*p* < 0.001 for all tissues).

Interaction analysis indicated that the CUR × OTC interaction did not significantly affect hepatic, renal, or splenic MDA concentrations, whereas a significant interaction was observed in gill tissue (Table 4, Table 5, Table 6 and Table 7). Thus, the reduction in MDA should be interpreted primarily as a general curcumin-associated effect in liver, kidney, and spleen, whereas a specific CUR × OTC interaction was evident only in gill tissue. Polynomial contrast analysis revealed predominantly linear reductions in MDA concentrations with increasing dietary curcumin supplementation, whereas significant quadratic and cubic responses were observed in selected tissues.

#### 3.3.2. Tissue SOD Activities

OTC treatment significantly influenced SOD activity in all examined tissues (Table 4, Table 5, Table 6 and Table 7). Increasing OTC concentration was accompanied by a progressive decline in SOD activity in the liver, kidney, spleen, and gills, with the lowest activities consistently observed in fish exposed to 75 mg/L OTC (main effect, *p* < 0.001 for all tissues).

Under normal rearing conditions, dietary curcumin supplementation resulted in progressively higher SOD activities in all examined tissues (Table 4, Table 5, Table 6 and Table 7). The highest SOD activities were generally recorded in fish receiving 100 mg/kg dietary curcumin.

Overall, dietary curcumin supplementation significantly increased SOD activity in all tissues examined (*p* < 0.001). Significant CUR × OTC interactions were observed in the liver and gills, whereas no significant interaction was detected in the kidney or spleen. Thus, the increase in SOD activity in the kidney and spleen appears to reflect the main effect of dietary curcumin primarily, while the significant interactions in the liver and gills indicate tissue-specific modulation of the response to OTC exposure. Polynomial contrast analysis further revealed predominantly linear increases in SOD activity with increasing dietary curcumin supplementation, although significant quadratic and cubic dose–response patterns were also detected in selected tissues.

#### 3.3.3. Tissue CAT Activities

CAT activity was significantly influenced by OTC treatment in all examined tissues (Table 4, Table 5, Table 6 and Table 7). OTC exposure resulted in a marked reduction in CAT activity, with the lowest activities generally observed in fish exposed to 75 mg/L OTC (main effect, *p* < 0.001). In the absence of OTC treatment, CAT activity increased with increasing dietary curcumin supplementation in all tissues, with the greatest activities generally recorded in fish fed 100 mg/kg curcumin. When all experimental groups were considered together, dietary curcumin supplementation significantly affected CAT activity in every tissue examined (*p* < 0.001). Significant CUR × OTC interactions were not detected in any tissue. Polynomial contrast analysis revealed predominantly linear responses to increasing dietary curcumin levels, whereas significant quadratic and cubic components were observed only in selected tissues.

#### 3.3.4. Tissue GSH-Px Activities

GSH-Px activity differed significantly among OTC treatments in all tissues examined (Table 4, Table 5, Table 6 and Table 7). Increasing OTC concentration was associated with progressively lower GSH-Px activity in the liver, kidney, spleen, and gills, with minimum activities consistently observed following exposure to 75 mg/L OTC (main effect, *p* < 0.001). Dietary curcumin supplementation produced a progressive increase in GSH-Px activity with increasing supplementation levels under both normal and OTC-treated conditions. Significant main effects of dietary curcumin were detected in all tissues (*p* < 0.001), whereas a significant CUR × OTC interaction was observed only in kidney tissue. Polynomial contrast analysis indicated predominantly linear responses to increasing dietary curcumin, with additional quadratic or cubic trends detected only in selected tissues.

#### 3.3.5. Tissue GST Activities

Unlike the remaining antioxidant parameters, GST activity increased significantly following OTC treatment in all tissues examined (Table 4, Table 5, Table 6 and Table 7). The highest GST activities were consistently recorded in fish exposed to 75 mg/L OTC (main effect, *p* < 0.001).

Dietary curcumin supplementation was associated with progressively lower GST activity in all tissues, and significant main effects of dietary curcumin were detected throughout the experiment (*p* < 0.001).

Significant CUR × OTC interactions were observed in the liver, kidney, and gills, whereas no interaction was detected in the spleen. Polynomial contrast analysis revealed predominantly linear responses to increasing dietary curcumin supplementation, while nonlinear responses were confined to selected tissues.

#### 3.3.6. Tissue GSH Levels

OTC treatment significantly reduced GSH concentrations in the liver, kidney, spleen, and gills (Table 4, Table 5, Table 6 and Table 7). Progressive declines in tissue GSH levels accompanied increasing OTC concentration, with the lowest concentrations consistently observed in fish receiving 75 mg/L OTC (main effect, *p* < 0.001 for all tissues).

Dietary curcumin supplementation significantly increased GSH concentrations in all examined tissues, both under normal conditions and following OTC treatment. Significant main effects of dietary curcumin were detected in every tissue (*p* < 0.001).

Interaction analysis revealed a significant CUR × OTC interaction only in gill tissue, whereas no interaction was detected in the liver, kidney, or spleen. Polynomial contrast analysis demonstrated predominantly linear responses to increasing dietary curcumin supplementation across all tissues.

## 4. Discussion

Hematological parameters are widely recognized as sensitive biomarkers for evaluating the physiological status and overall health of fish, particularly under chemical or environmental stress. In the present study, OTC exposure significantly reduced RBC count, Hb concentration, Ht, MCV, and MCH, indicating impaired erythrocyte homeostasis and oxygen-carrying capacity in rainbow trout. Similar hematological alterations have been reported in rainbow trout [33], Nile tilapia [34], common carp [35], and pangasius (*Pangasianodon hypophthalmus*) [36], suggesting that hematological impairment is a common response to OTC exposure across teleost species. These changes may be largely attributed to OTC-induced oxidative stress, as excessive ROS generation can promote lipid peroxidation of erythrocyte membranes, increase erythrocyte fragility, and impair hematopoietic activity. Such effects may ultimately contribute to the observed reductions in erythrocyte-related indices and compromise blood oxygen transport. Moreover, the progressively greater hematological alterations observed at higher OTC concentrations are consistent with previous evidence showing that increasing OTC exposure can intensify physiological disturbances, histopathological damage, and systemic toxicity in cultured fish [34]. Unlike the other erythrocyte indices, MCHC exhibited a non-monotonic response to OTC exposure, with a slight increase at 50 mg/L followed by a decrease at 75 mg/L. This pattern suggests that MCHC may be less sensitive to graded OTC exposure than RBC, Hb, Ht, MCV, and MCH and may reflect a distinct hematological response to OTC exposure, consistent with previously reported alterations in erythrocyte-related indices in fish exposed to OTC [35,37].

Dietary curcumin supplementation alone significantly improved the hematological profile of rainbow trout in the present study, as evidenced by progressive increases in RBC count, Hb concentration, Ht, MCV, and MCH. Similar improvements in erythrocyte-related parameters have been reported in rainbow trout fed curcumin-supplemented diets, where increases in RBC, Hb, and Ht were accompanied by enhanced physiological performance and disease resistance [38]. Comparable hematological improvements have also been reported in common carp [39], Nile tilapia [40], and European seabass [41], indicating that the beneficial effects of dietary curcumin on hematological function are consistently observed across cultured fish species.

The beneficial hematological effects of curcumin are likely related to its antioxidant, membrane-protective, and hematopoietic properties. Curcumin can reduce ROS-mediated lipid peroxidation and preserve erythrocyte membrane integrity, thereby limiting erythrocyte fragility [42], while maintenance of intracellular antioxidant defenses and redox homeostasis may further support hematopoietic function [43]. In the present study, dietary curcumin alleviated OTC-induced hematological disturbances, with the greatest overall recovery generally observed at the highest inclusion level. Although direct evidence on curcumin against OTC-induced hematological toxicity remains limited, similar protective effects of antioxidant supplementation against antibiotic-related hematological impairment have been reported in fish [33,34]. The present findings are also supported by evidence that curcumin can ameliorate hematological alterations induced by oxidative toxicants such as lead and carbofuran through attenuation of oxidative damage and reinforcement of endogenous antioxidant defenses [39,44]. Collectively, these observations suggest that the recovery of erythrocyte-related indices in OTC-exposed rainbow trout is associated with the antioxidant and cytoprotective actions of dietary curcumin.

The innate immune system constitutes the primary defense mechanism in fish, providing rapid protection against invading pathogens through coordinated cellular and humoral immune responses. Accordingly, the present study demonstrated that OTC exposure markedly suppressed innate immune function. These findings indicate that OTC compromises both cellular and humoral branches of the innate immune system in rainbow trout.

The immunosuppressive effects observed in the present study closely align with numerous previous investigations. Early studies in common carp demonstrated that OTC suppresses both cellular and humoral immune responses, resulting in impaired immune competence [45]. Similar reductions in innate immune activity have subsequently been reported in rainbow trout [33], gilthead seabream [46], Nile tilapia [47], European seabass [48], and Atlantic salmon immune cells [49]. Collectively, these studies demonstrate that suppression of innate immunity represents one of the most consistent physiological consequences of excessive OTC exposure in teleost fish. The observed immunosuppression is primarily attributed to OTC-induced oxidative stress, which disrupts leukocyte integrity, impairs phagocytic activity and respiratory burst capacity, alters the expression of immune-related genes, and suppresses both cellular and humoral immune responses, thereby increasing susceptibility to opportunistic pathogens [46,48]. Therefore, the dose-dependent decline in immune parameters observed in the present study most likely reflects progressive impairment of leukocyte function and antimicrobial defense during prolonged OTC exposure.

Dietary curcumin supplementation enhanced both cellular and humoral innate immune responses in rainbow trout, consistent with previous reports of its immunostimulatory effects in rainbow trout [38], Nile tilapia [40,50,51,52], common carp [53], grass carp [54], snakehead fish [55], and European seabass [41]. Collectively, these findings support the broad immunomodulatory potential of dietary curcumin across cultured fish species.

The immunostimulatory effects of curcumin are likely related to its combined antioxidant, anti-inflammatory, and immune-modulatory activities. Curcumin can enhance leukocyte function, phagocytic activity, respiratory burst, and humoral immune responses [38,40,50]. These effects may also involve activation of Nrf2-dependent antioxidant signaling and suppression of NF-κB-mediated inflammation, thereby supporting immune-related gene expression and limiting pro-inflammatory responses [51,54,55,56]. Additional regulation of miR-155 and Th17-cell differentiation may help maintain immune homeostasis [57,58], while preserving intracellular redox balance helps sustain leukocyte integrity and antimicrobial function under stress [59]. In the present study, dietary curcumin progressively alleviated OTC-induced suppression of cellular and humoral immune responses. Although direct evidence for its protective effect against OTC-induced immunosuppression is limited, similar immunoprotective effects have been reported under heavy-metal, pesticide, bacterial, and other oxidative stress-related challenges in fish [39,52]. Collectively, these findings suggest that curcumin may contribute to improved immune status under OTC exposure, although the response was not statistically uniform across all immune endpoints.

Oxidative stress is one of the principal mechanisms underlying antibiotic-induced toxicity in fish and reflects an imbalance between reactive oxygen species (ROS) generation and endogenous antioxidant capacity. In the present study, OTC exposure induced a marked disturbance of redox homeostasis in rainbow trout, as demonstrated by increased MDA concentrations accompanied by reductions in SOD, CAT, GSH-Px, and GSH in the liver, kidney, spleen, and gills. By contrast, GST activity increased with OTC concentration, indicating the induction of a compensatory detoxification response under elevated oxidative pressure. Collectively, these findings show that OTC promotes lipid peroxidation while weakening major enzymatic and non-enzymatic antioxidant defenses in multiple tissues. The oxidative alterations recorded in this study are consistent with previous evidence demonstrating that OTC induces redox imbalance in a range of aquatic organisms. Increased lipid peroxidation together with altered or depleted antioxidant defenses has been reported in rainbow trout [60], common carp [61], Nile tilapia [34], grass carp [62], freshwater stinging catfish [63], and Atlantic salmon liver and gill cell cultures [64,65]. Moreover, antioxidant supplementation in silver catfish further supports the central role of oxidative stress in OTC toxicity by attenuating OTC-induced oxidative damage [66]. Together, these observations indicate that disruption of redox regulation is a recurring physiological consequence of OTC exposure in aquatic organisms.

OTC-induced oxidative stress appears to involve excessive ROS generation, mitochondrial dysfunction, and weakening of antioxidant defenses. Impaired mitochondrial dynamics and electron transport can increase ROS production and promote oxidative damage to lipids, proteins, and DNA [62,67]. Accordingly, the increased MDA levels observed in the present study likely reflect enhanced lipid peroxidation, whereas the reductions in SOD, CAT, GSH-Px, and GSH may result from progressive utilization, inactivation, or depletion during sustained oxidative challenge [60,61,63]. The increase in GST may represent a compensatory phase II detoxification response, although this adaptation was insufficient to restore overall redox balance. In addition, tetracycline antibiotics may interfere with Nrf2-regulated signaling and further compromise cellular antioxidant regulation [64,68].

In contrast, dietary curcumin improved antioxidant status in rainbow trout, as reflected by lower MDA and higher SOD, CAT, GSH-Px, and GSH levels, while GST showed a tissue-dependent response. Similar antioxidant effects of curcumin have been reported in Nile tilapia [40,51], common carp [69], largemouth bass [70], gibel carp [71], and grass carp [72]. Comparable protective effects have also been observed under exposure to fipronil, chromium, ammonia, and other oxidative stressors [52,73]. Collectively, these findings support the antioxidant and redox-modulating role of dietary curcumin in fish.

The antioxidant activity of curcumin involves both direct and signaling-mediated mechanisms. Curcumin can neutralize reactive species directly while enhancing endogenous defenses by activating Nrf2-dependent transcription, thereby promoting cytoprotective systems associated with SOD, CAT, GSH-Px, GST, HO-1, NQO1, and glutathione metabolism [74,75]. Curcumin may also reduce intracellular ROS accumulation, preserve mitochondrial function, alleviate endoplasmic reticulum stress, and limit apoptosis and ferroptosis under oxidative challenge [70,71]. Through these complementary actions, curcumin can restrict membrane lipid peroxidation, preserve intracellular glutathione availability, and maintain the functional capacity of enzymatic antioxidant defenses. This mechanistic framework plausibly explains the lower MDA concentrations and improved SOD, CAT, GSH-Px, and GSH responses observed in healthy rainbow trout receiving curcumin-supplemented diets.

More importantly, dietary curcumin substantially mitigated OTC-induced oxidative disturbances by reducing tissue MDA concentrations, improving SOD, CAT, GSH-Px, and GSH responses, and attenuating the OTC-associated increase in GST activity. These effects likely reflect both direct antioxidant activity and modulation of redox-sensitive signaling pathways [75,76]. In particular, curcumin may enhance Nrf2/ARE signaling by promoting Nrf2 activity and reducing Keap1-mediated inhibition, thereby strengthening endogenous antioxidant and glutathione-dependent defenses [67,77,78]. Curcumin may also preserve mitochondrial integrity, reduce endoplasmic reticulum stress, and limit oxidative stress-related apoptosis and ferroptosis [67,75]. In parallel, suppressing NF-κB-mediated inflammatory signaling and activating Nrf2/HO-1-dependent cytoprotection may further reduce the interaction between inflammation and oxidative stress [79,80]. Collectively, these mechanisms provide a plausible explanation for the improved redox balance observed in OTC-exposed rainbow trout.

Several limitations of the present study should be acknowledged. Biomarker assessments were performed at the individual-fish level, and the statistical model did not account for potential tank-level variability. In addition, OTC concentrations in the exposure water and its accumulation in fish tissues were not directly measured, limiting interpretation of exposure dynamics and preventing determination of whether the observed protective effects of curcumin were related to altered OTC bioaccumulation or primarily to modulation of OTC-induced physiological and oxidative disturbances. Consequently, possible changes in OTC concentration during the 48-h exposure period due to degradation were not quantified, and the potential influence of water temperature and pH on OTC stability was not specifically assessed. The relatively low oral bioavailability of curcumin may also have influenced its absorption and biological efficacy. Furthermore, the curcumin concentrations in the finished pelleted diets were not analytically verified after feed preparation; therefore, the dietary curcumin levels reported in this study represent nominal inclusion levels based on the amounts added during diet formulation. Possible losses or alterations in curcumin concentration during pelleting, drying, and storage could not be quantified. Although no overt adverse effects were observed during the 8-week feeding period, the long-term safety of prolonged curcumin supplementation was not specifically evaluated. Furthermore, because the study was conducted in healthy fish, the potential influence of curcumin on the antibacterial efficacy of OTC during infection was not assessed. Future studies should therefore incorporate tank-level statistical effects, quantify OTC in water and tissues, evaluate curcumin pharmacokinetics and long-term safety, and investigate possible curcumin–OTC interactions under clinically relevant bacterial challenge conditions.

## 5. Conclusions

This study demonstrated that dietary curcumin alleviated OTC-induced physiological disturbances in rainbow trout by improving hematological, immune, and antioxidant responses. OTC exposure impaired erythrocyte homeostasis, suppressed innate immunity, and disrupted redox balance, whereas curcumin supplementation improved these parameters under both normal and OTC-challenged conditions. Increasing dietary curcumin generally enhanced recovery of erythrocyte-related indices, innate immune responses, and antioxidant status, while reducing lipid peroxidation. These protective effects are likely associated with the antioxidant, anti-inflammatory, and immunomodulatory properties of curcumin and its ability to support redox homeostasis.

Overall, dietary curcumin was associated with improvements in hematological, immune, and antioxidant parameters in OTC-exposed rainbow trout, with the 100 mg/kg feed level producing the most favorable overall response among the tested inclusion levels. These findings support further evaluation of dietary curcumin as a functional feed additive; however, additional studies are needed to determine its long-term safety, bioavailability, effects on disease resistance and survival, and potential interactions with OTC efficacy under aquaculture conditions.

## Figures and Tables

**Table 1:** Effects of dietary curcumin supplementation against oxytetracycline-induced hematological alterations in rainbow trout.

		RBC	Hb	Ht	MCV	MCH	MCHC
OTC 0 mg/L		1.67 ± 0.08 ^x^	7.80 ± 0.63 ^x^	36.83 ± 3.01 ^x^	219.73 ± 7.47 ^x^	46.56 ± 1.65 ^x^	21.19 ± 0.46 ^x^
OTC 50 mg/L		1.50 ± 0.09 ^y^	5.96 ± 0.57 ^y^	27.23 ± 2.80 ^y^	180.55 ± 8.87 ^y^	39.55 ± 1.67 ^y^	21.92 ± 0.41 ^y^
OTC 75 mg/L		1.40 ± 0.08 ^z^	5.15 ± 0.59 ^z^	25.00 ± 3.97 ^z^	177.68 ± 19.02 ^z^	36.65 ± 2.30 ^z^	20.74 ± 1.19 ^z^
						
CUR 0 mg/kg		1.41 ± 0.10 ^D^	5.51 ± 1.12 ^D^	25.28 ± 5.25 ^D^	177.75 ± 23.43 ^D^	38.78 ± 4.96 ^D^	21.83 ± 0.49 ^A^
CUR 10 mg/kg		1.51 ± 0.13 ^C^	6.11 ± 1.13 ^C^	28.39 ± 6.06 ^C^	186.13 ± 23.64 ^C^	40.14 ± 3.99 ^C^	21.64 ± 0.74 ^A^
CUR 50 mg/kg		1.57 ± 0.13 ^B^	6.61 ± 1.20 ^B^	31.60 ± 4.96 ^B^	200.20 ± 16.62 ^B^	41.78 ± 4.24 ^B^	20.86 ± 0.85 ^B^
CUR 100 mg/kg		1.61 ± 0.11 ^A^	6.98 ± 1.15 ^A^	33.48 ± 4.85 ^A^	206.54 ± 16.55 ^A^	42.99 ± 4.13 ^A^	20.81 ± 1.01 ^B^
						
OTC 0	CUR 0	1.55 ± 0.02 ^d^	6.98 ± 0.21	32.32 ± 0.45 ^d^	209.23 ± 1.18 ^d^	45.15 ± 0.82 ^b^	21.58 ± 0.38 ^c,d,e^
	CUR 10	1.67 ± 0.03 ^b^	7.56 ± 0.25	36.43 ± 0.70 ^c^	218.03 ± 1.91 ^c^	45.25 ± 0.91 ^b^	20.75 ± 0.30 ^f^
	CUR 50	1.72 ± 0.02 ^a^	8.19 ± 0.21	38.38 ± 0.68 ^b^	222.62 ± 1.91 ^b^	47.52 ± 0.70 ^a^	21.34 ± 0.31 ^d,e^
	CUR 100	1.75 ± 0.02 ^a^	8.48 ± 0.29	40.18 ± 0.62 ^a^	229.05 ± 1.33 ^a^	48.35 ± 1.08 ^a^	21.11 ± 0.40 ^e,f^
OTC 50	CUR 0	1.38 ± 0.02 ^g^	5.18 ± 0.18	23.26 ± 0.56 ^i^	169.02 ± 1.63 ^i^	37.65 ± 0.77 ^f^	22.28 ± 0.30 ^a^
	CUR 10	1.49 ± 0.02 ^e^	5.84 ± 0.21	26.38 ± 0.62 ^h^	176.58 ± 1.93 ^h^	39.06 ± 0.84 ^d,e^	22.12 ± 0.32 ^a,b^
	CUR 50	1.56 ± 0.02 ^c,d^	6.22 ± 0.19	28.93 ± 0.74 ^f^	184.92 ± 2.66 ^g^	39.73 ± 0.68 ^d^	21.49 ± 0.24 ^d,e^
	CUR 100	1.58 ± 0.02 ^c^	6.62 ± 0.20	30.36 ± 0.61 ^e^	191.67 ± 1.81 ^f^	41.76 ± 0.80 ^c^	21.79 ± 0.26 ^a,b,c,d^
OTC 75	CUR 0	1.31 ± 0.02 ^h^	4.38 ± 0.20	20.25 ± 0.61 ^j^	155.01 ± 2.18 ^k^	33.53 ± 1.06 ^h^	21.63 ± 0.46 ^b,c,d^
	CUR 10	1.36 ± 0.02 ^g^	4.93 ± 0.23	22.34 ± 0.61 ^i^	163.77 ± 2.47 ^j^	36.10 ± 1.16 ^g^	22.04 ± 0.50 ^a,b,c^
	CUR 50	1.42 ± 0.02 ^f^	5.43 ± 0.20	27.49 ± 0.64 ^g^	193.06 ± 2.12 ^f^	38.11 ± 0.90 ^e,f^	19.74 ± 0.30 ^g^
	CUR 100	1.50 ± 0.02 ^e^	5.85 ± 0.21	29.92 ± 0.65 ^e^	198.89 ± 1.93 ^e^	38.86 ± 0.86 ^d,e,f^	19.54 ± 0.33 ^g^
------------------------------------------------------------*p *values----------------------------------------------------------							
OTC		<0.001	<0.001	<0.001	<0.001	<0.001	<0.001
CUR		<0.001	<0.001	<0.001	<0.001	<0.001	<0.001
OTC × CUR		<0.001	0.609	<0.001	<0.001	<0.001	<0.001
CUR effect for	Contrast---------------------------------------------------------------------------------------------------------						
OTC 0 mg/L	Linear	<0.001	<0.001	<0.001	<0.001	<0.001	<0.001
	Quadratic	<0.001	0.059	<0.001	0.026	0.203	0.011
	Cubic	0.950	0.264	0.028	0.012	0.007	<0.001
OTC 50 mg/L	Linear	<0.001	<0.001	<0.001	<0.001	<0.001	<0.001
	Quadratic	<0.001	0.046	<0.001	0.591	0.214	0.014
	Cubic	0.144	0.283	0.567	0.420	0.063	0.001
OTC 75 mg/L	Linear	<0.001	<0.001	<0.001	<0.001	<0.001	<0.001
	Quadratic	0.126	0.350	0.413	0.410	0.007	0.023
	Cubic	0.570	0.892	<0.001	<0.001	0.629	<0.001

RBC: Red blood cell (×10^6^); Hb: Hemoglobin (g/dL); Ht: Hematocrit (%); MCV: Mean corpuscular volume (µm^3^); MCH: Mean corpuscular hemoglobin (pg); MCHC: Mean corpuscular hemoglobin concentration (%). Data are presented as mean and standard deviation (*n* = 30 fish per treatment combination). ^x,y,z^ Differences between means indicated by different letters in the same column are statistically significant for the OTC main effect (*p* < 0.05). ^A,B,C,D^ Differences between means indicated by different capital letters in the same column are statistically significant for CUR main effect (*p* < 0.05). ^a,b,c,d,e,f,g,h,i,j,k^ Differences between means indicated by different letters in the same column are statistically significant for OTC × CUR interactions (*p* < 0.05).

**Table 2:** Effects of dietary curcumin supplementation against oxytetracycline-induced alterations on white blood cell counts, nitroblue tetrazolium assay, phagocytic activity, phagocytic index, and total protein level of rainbow trout.

		WBC	NBT	PA	PI	TP
OTC 0 mg/L		40.94 ± 3.44 ^x^	1.38 ± 0.14 ^x^	1.37 ± 0.18 ^x^	3.73 ± 0.34 ^x^	30.98 ± 5.13 ^x^
OTC 50 mg/L		32.13 ± 2.71 ^y^	0.97 ± 0.08 ^y^	1.00 ± 0.17 ^y^	3.17 ± 0.33 ^y^	22.53 ± 4.54 ^y^
OTC 75 mg/L		29.41 ± 3.10 ^z^	0.85 ± 0.11 ^z^	0.89 ± 0.17 ^z^	3.04 ± 0.28 ^y^	20.01 ± 3.54 ^z^
					
CUR 0 mg/kg		29.99 ± 4.80 ^D^	0.92 ± 0.20 ^D^	0.89 ± 0.25 ^D^	3.09 ± 0.44 ^C^	19.76 ± 4.63 ^D^
CUR 10 mg/kg		32.85 ± 4.76 ^C^	1.04 ± 0.23 ^C^	1.03 ± 0.21 ^C^	3.26 ± 0.37 ^B,C^	22.52 ± 4.86 ^C^
CUR 50 mg/kg		35.88 ± 5.40 ^B^	1.12 ± 0.26 ^B^	1.17 ± 0.24 ^B^	3.44 ± 0.39 ^A,B^	26.91 ± 6.41 ^B^
CUR 100 mg/kg		37.93 ± 5.16 ^A^	1.19 ± 0.24 ^A^	1.27 ± 0.23 ^A^	3.47 ± 0.45 ^A^	28.84 ± 5.68 ^A^
					
OTC 0	CUR 0	36.36 ± 0.46 ^d^	1.19 ± 0.02 _d_	1.21 ± 0.13	3.56 ± 0.34	25.78 ± 1.92
	CUR 10	39.22 ± 0.41 ^c^	1.34 ± 0.02 ^c^	1.28 ± 0.12	3.60 ± 0.37	28.19 ± 3.40
	CUR 50	43.23 ± 0.46 ^b^	1.48 ± 0.02 ^b^	1.45 ± 0.15	3.85 ± 0.24	34.62 ± 3.52
	CUR 100	44.96 ± 0.41 ^a^	1.53 ± 0.02 ^a^	1.56 ± 0.09	3.93 ± 0.29	35.31 ± 3.50
OTC 50	CUR 0	28.44 ± 0.45 ^h^	0.86 ± 0.02 ^h^	0.78 ± 0.06	2.87 ± 0.27	17.06 ± 1.54
	CUR 10	31.15 ± 0.46 ^g^	0.97 ± 0.02 ^g^	0.97 ± 0.08	3.12 ± 0.23	20.77 ± 2.25
	CUR 50	33.39 ± 0.47 ^f^	1.02 ± 0.02 ^f^	1.12 ± 0.11	3.30 ± 0.28	25.21 ± 2.41
	CUR 100	35.54 ± 0.39 ^e^	1.06 ± 0.02 ^e^	1.15 ± 0.11	3.39 ± 0.30	27.09 ± 2.89
OTC 75	CUR 0	25.17 ± 0.45 ^i^	0.72 ± 0.02 ^j^	0.69 ± 0.07	2.83 ± 0.28	16.43 ± 1.49
	CUR 10	28.18 ± 0.33 ^h^	0.81 ± 0.02 ^i^	0.84 ± 0.09	3.05 ± 0.25	18.61 ± 1.80
	CUR 50	31.03 ± 0.46 ^g^	0.87 ± 0.02 ^h^	0.95 ± 0.09	3.18 ± 0.27	20.90 ± 2.17
	CUR 100	33.27 ± 0.34 ^f^	1.00 ± 0.02 ^f,g^	1.09 ± 0.10	3.08 ± 0.25	24.10 ± 2.89
------------------------------------------------------------*p *values----------------------------------------------------------						
OTC		<0.001	<0.001	<0.001	<0.001	<0.001
CUR		<0.001	<0.001	<0.001	<0.001	<0.001
OTC × CUR		<0.001	<0.001	0.262	0.591	0.109
CUR effect for	Contrast---------------------------------------------------------------------------------------------------------					
OTC 0 mg/L	Linear	<0.001	<0.001	<0.001	0.004	<0.001
	Quadratic	<0.001	<0.001	0.576	0.873	0.394
	Cubic	<0.001	0.013	0.365	0.380	0.035
OTC 50 mg/L	Linear	<0.001	<0.001	<0.001	<0.001	<0.001
	Quadratic	0.056	<0.001	0.006	0.374	0.223
	Cubic	0.541	0.129	0.613	0.949	0.325
OTC 75 mg/L	Linear	<0.001	<0.001	<0.001	0.023	<0.001
	Quadratic	0.005	0.008	0.971	0.061	0.454
	Cubic	0.437	0.004	0.491	0.685	0.796

WBC: White blood cell counts (×103); NBT: Nitroblue tetrazolium assay (mg/mL); PA: Phagocytic activity (%); PI: Phagocytic index; TP: Total protein level (mg/mL). Data are presented as mean and standard deviation (*n* = 30 fish per treatment combination). ^x,y,z^ Differences between means indicated by different letters in the same column are statistically significant for the OTC main effect (*p* < 0.05). ^A,B,C,D^ Differences between means indicated by different capital letters in the same column are statistically significant for CUR main effect (*p* < 0.05). ^a,b,c,d,e,f,g,h,i,j^ Differences between means indicated by different letters in the same column are statistically significant for OTC × CUR interactions (*p* < 0.05).

**Table 3:** Effects of dietary curcumin supplementation against oxytetracycline-induced alterations on total immunoglobulin M level, serum bactericidal activity, lysozyme activity, and myeloperoxidase activity of rainbow trout.

		IgM	BA	LYZ	MPO
OTC 0 mg/L		18.32 ± 3.29 ^x^	58.39 ± 8.45 ^x^	157.1 ± 19.74 ^x^	0.97 ± 0.16 ^x^
OTC 50 mg/L		12.28 ± 1.92 ^y^	40.62 ± 5.32 ^y^	109.27 ± 15.37 ^y^	0.63 ± 0.12 ^y^
OTC 75 mg/L		11.34 ± 1.78 ^z^	37.58 ± 5.21 ^z^	94.72 ± 13.93 ^z^	0.57 ± 0.12 ^z^
				
CUR 0 mg/kg		11.16 ± 2.27 ^D^	38.52 ± 7.61 ^D^	100.03 ± 24.01 ^D^	0.57 ± 0.18 ^D^
CUR 10 mg/kg		13.39 ± 3.23 ^C^	43.44 ± 9.38 ^C^	116.41 ± 29.35 ^C^	0.67 ± 0.17 ^C^
CUR 50 mg/kg		14.63 ± 3.66 ^B^	47.99 ± 11.45 ^B^	129.08 ± 30.58 ^B^	0.80 ± 0.20 ^B^
CUR 100 mg/kg		16.75 ± 4.17 ^A^	52.17 ± 11.62 ^A^	135.93 ± 29.92 ^A^	0.85 ± 0.20 ^A^
				
OTC 0	CUR 0	14.09 ± 1.14 ^d^	47.92 ± 3.73 ^c^	130.29 ± 9.27 ^c^	0.80 ± 0.10
	CUR 10	17.56 ± 1.29 ^c^	55.87 ± 3.20 ^b^	156.06 ± 7.02 ^b^	0.89 ± 0.08
	CUR 50	19.40 ± 1.44 ^b^	62.76 ± 4.61 ^a^	167.87 ± 11.17 ^a,b^	1.06 ± 0.09
	CUR 100	22.26 ± 1.65 ^a^	67.02 ± 5.61 ^a^	174.18 ± 13.28 ^a^	1.12 ± 0.10
OTC 50	CUR 0	9.89 ± 0.80 ^g,h^	35.73 ± 3.42 ^e,f^	93.95 ± 6.02 ^f^	0.48 ± 0.05
	CUR 10	12.02 ± 1.17 ^e,f^	38.31 ± 2.99 ^d,e^	98.92 ± 8.77 ^e,f^	0.59 ± 0.07
	CUR 50	13.06 ± 1.04 ^d,e^	42.36 ± 4.34 ^c,d^	120.17 ± 8.42 ^c,d^	0.70 ± 0.06
	CUR 100	14.16 ± 1.39 ^d^	46.08 ± 3.80 ^c^	124.03 ± 9.25 ^c^	0.75 ± 0.06
OTC 75	CUR 0	9.49 ± 0.46 ^h^	31.92 ± 2.43 ^f^	75.85 ± 5.20 ^g^	0.42 ± 0.04
	CUR 10	10.59 ± 0.72 ^f,g,h^	36.14 ± 2.08 ^e,f^	94.26 ± 4.09 ^f^	0.54 ± 0.05
	CUR 50	11.44 ± 0.86 ^e,f,g^	38.85 ± 3.48 ^d,e^	99.20 ± 8.08 ^e,f^	0.64 ± 0.06
	CUR 100	13.85 ± 0.91 ^d^	43.43 ± 4.21 ^c,d^	109.58 ± 8.22 ^d,e^	0.70 ± 0.06
------------------------------------------------------------*p *values----------------------------------------------------------					
OTC		<0.001	<0.001	<0.001	<0.001
CUR		<0.001	<0.001	<0.001	<0.001
OTC × CUR		<0.001	0.003	0.002	0.610
CUR effect for	Contrast---------------------------------------------------------------------------------------------------------				
OTC 0 mg/L	Linear	<0.001	<0.001	<0.001	<0.001
	Quadratic	0.493	0.190	0.006	0.534
	Cubic	0.186	0.802	0.570	0.129
OTC 50 mg/L	Linear	<0.001	<0.001	<0.001	<0.001
	Quadratic	0.154	0.629	0.831	0.088
	Cubic	0.483	0.730	0.006	0.586
OTC 75 mg/L	Linear	<0.001	<0.001	<0.001	<0.001
	Quadratic	0.010	0.858	0.064	0.104
	Cubic	0.100	0.456	0.052	0.736

IgM: Total immunoglobulin M level (mg/mL); BA: Serum bactericidal activity (% cfu/control); LYZ: Lysozyme activity (U/mL); MPO: Myeloperoxidase activity (Optical density). Data are presented as mean and standard deviation (*n* = 30 fish per treatment combination). ^x,y,z^ Differences between means indicated by different letters in the same column are statistically significant for OTC main effect (*p* < 0.05). ^A,B,C,D^ Differences between means indicated by different capital letters in the same column are statistically significant for CUR main effect (*p* < 0.05). ^a,b,c,d,e,f,g,h^ Differences between means indicated by different letters in the same column are statistically significant for OTC × CUR interactions (*p* < 0.05).

**Table 4:** Effects of dietary curcumin supplementation against oxytetracycline-induced oxidative stress and antioxidant alterations in the liver of rainbow trout.

Liver		MDA	SOD	CAT	GSH-Px	GST	GSH
OTC 0 mg/L		4.33 ± 0.51 ^z^	3.57 ± 0.40 ^x^	4.02 ± 0.52 ^x^	3.37 ± 0.43 ^x^	143.99 ± 11.26 ^z^	61.71 ± 7.67 ^x^
OTC 50 mg/L		5.59 ± 0.69 ^y^	2.70 ± 0.42 ^y^	2.77 ± 0.40 ^y^	2.56 ± 0.32 ^y^	164.08 ± 17.74 ^y^	40.33 ± 7.06 ^y^
OTC 75 mg/L		6.42 ± 0.61 ^x^	2.42 ± 0.45 ^z^	2.69 ± 0.39 ^y^	2.25 ± 0.32 ^z^	178.95 ± 21.37 ^x^	38.43 ± 6.16 ^y^
						
CUR 0 mg/kg		6.05 ± 0.95 ^A^	2.44 ± 0.59 ^C^	2.73 ± 0.67 ^D^	2.33 ± 0.46 ^C^	175.76 ± 28.47 ^A^	38.73 ± 10.79 ^D^
CUR 10 mg/kg		5.52 ± 1.08 ^B^	2.81 ± 0.65 ^B^	3.06 ± 0.59 ^C^	2.71 ± 0.59 ^B^	166.51 ± 22.77 ^B^	45.93 ± 13.10 ^C^
CUR 50 mg/kg		5.23 ± 1.01 ^B,C^	3.08 ± 0.52 ^A^	3.31 ± 0.75 ^B^	2.92 ± 0.61 ^A^	157.17 ± 14.56 ^C^	49.60 ± 11.10 ^B^
CUR 100 mg/kg		5.00 ± 0.91 ^C^	3.25 ± 0.52 ^A^	3.54 ± 0.76 ^A^	2.94 ± 0.52 ^A^	149.93 ± 11.18 ^D^	53.04 ± 11.41 ^A^
						
OTC 0	CUR 0	4.91 ± 0.50	3.19 ± 0.31 ^b,c^	3.58 ± 0.35	2.85 ± 0.33	140.32 ± 9.48 ^c^	53.02 ± 3.78
	CUR 10	4.27 ± 0.26	3.60 ± 0.34 ^a,b^	3.77 ± 0.28	3.42 ± 0.25	139.97 ± 11.23 ^c^	62.93 ± 7.30
	CUR 50	4.14 ± 0.45	3.67 ± 0.26 ^a^	4.27 ± 0.39	3.68 ± 0.31	146.11 ± 10.58 ^c^	63.72 ± 5.25
	CUR 100	4.00 ± 0.26	3.80 ± 0.43 ^a^	4.47 ± 0.47	3.54 ± 0.33	149.57 ± 12.21 ^c^	67.19 ± 6.02
OTC 50	CUR 0	6.36 ± 0.51	2.15 ± 0.18 ^g^	2.30 ± 0.26	2.21 ± 0.24	185.55 ± 11.70 ^a,b^	32.67 ± 3.61
	CUR 10	5.66 ± 0.46	2.64 ± 0.20 ^d,e^	2.79 ± 0.29	2.58 ± 0.28	171.10 ± 9.22 ^b^	36.75 ± 2.76
	CUR 50	5.22 ± 0.48	2.98 ± 0.28 ^c,d,e^	2.88 ± 0.25	2.70 ± 0.25	154.33 ± 6.07 ^c^	44.88 ± 3.59
	CUR 100	5.13 ± 0.53	3.02 ± 0.29 ^c,d^	3.10 ± 0.32	2.74 ± 0.25	145.35 ± 6.52 ^c^	47.01 ± 5.49
OTC 75	CUR 0	6.87 ± 0.29	1.97 ± 0.18 ^g^	2.31 ± 0.25	1.94 ± 0.21	201.41 ± 12.29 ^a^	30.51 ± 2.27
	CUR 10	6.63 ± 0.62	2.18 ± 0.22 ^f,g^	2.61 ± 0.29	2.15 ± 0.25	188.47 ± 10.88 ^a^	38.12 ± 3.15
	CUR 50	6.33 ± 0.42	2.59 ± 0.26 ^e,f^	2.80 ± 0.31	2.39 ± 0.19	171.07 ± 13.23 ^b^	40.20 ± 3.52
	CUR 100	5.87 ± 0.59	2.93 ± 0.32 ^c,d,e^	3.03 ± 0.31	2.53 ± 0.26	154.87 ± 12.67 ^c^	44.91 ± 4.15
------------------------------------------------------------*p *values----------------------------------------------------------							
OTC		<0.001	<0.001	<0.001	<0.001	<0.001	<0.001
CUR		<0.001	<0.001	<0.001	<0.001	<0.001	<0.001
OTC × CUR		0.324	0.033	0.394	0.160	<0.001	0.181
CUR effect for	Contrast---------------------------------------------------------------------------------------------------------						
OTC 0 mg/L	Linear	<0.001	<0.001	<0.001	<0.001	0.035	<0.001
	Quadratic	0.048	0.180	0.947	<0.001	0.586	0.084
	Cubic	0.324	0.411	0.271	0.834	0.556	0.155
OTC 50 mg/L	Linear	<0.001	<0.001	<0.001	<0.001	<0.001	<0.001
	Quadratic	0.060	0.005	0.146	0.049	0.326	0.445
	Cubic	0.881	0.681	0.168	0.629	0.416	0.083
OTC 75 mg/L	Linear	<0.001	<0.001	<0.001	<0.001	<0.001	<0.001
	Quadratic	0.506	0.395	0.724	0.691	0.678	0.177
	Cubic	0.881	0.445	0.699	0.686	0.747	0.093

MDA: Malondialdehyde (nmol/mg protein); SOD: Superoxide dismutase (U/mg protein); CAT: Catalase (k/mg protein); GSH-Px: Glutathione peroxidase (U/mg protein); GST: Glutathione S-transferase (U/mg protein); GSH: Reduced glutathione (µmol/mg protein). Data are presented as mean and standard deviation (*n* = 30 fish per treatment combination). ^x,y,z^ Differences between means indicated by different letters in the same column are statistically significant for the OTC main effect (*p* < 0.05). ^A,B,C,D^ Differences between means indicated by different capital letters in the same column are statistically significant for CUR main effect (*p* < 0.05). ^a,b,c,d,e,f,g^ Differences between means indicated by different letters in the same column are statistically significant for OTC × CUR interactions (*p* < 0.05).

**Table 5:** Effects of dietary curcumin supplementation against oxytetracycline-induced oxidative stress and antioxidant alterations in the kidney of rainbow trout.

Kidney		MDA	SOD	CAT	GSH-Px	GST	GSH
OTC 0 mg/L		3.12 ± 0.68 ^z^	3.32 ± 0.51 ^x^	3.23 ± 0.40 ^x^	3.42 ± 0.59 ^x^	123.10 ± 11.38 ^z^	55.33 ± 7.88 ^x^
OTC 50 mg/L		5.09 ± 0.65 ^y^	2.50 ± 0.40 ^y^	2.44 ± 0.30 ^y^	2.29 ± 0.32 ^y^	162.59 ± 16.53 ^y^	37.04 ± 5.63 ^y^
OTC 75 mg/L		5.67 ± 0.83 ^x^	2.18 ± 0.37 ^z^	2.16 ± 0.34 ^z^	2.09 ± 0.31 ^z^	176.81 ± 22.67 ^x^	33.27 ± 6.07 ^z^
						
CUR 0 mg/kg		5.45 ± 1.17 ^A^	2.16 ± 0.46 ^C^	2.21 ± 0.50 ^C^	2.20 ± 0.54 ^D^	173.50 ± 33.50 ^A^	35.46 ± 10.32 ^D^
CUR 10 mg/kg		4.84 ± 1.28 ^B^	2.53 ± 0.48 ^B^	2.60 ± 0.44 ^B^	2.41 ± 0.46 ^C^	158.58 ± 26.18 ^B^	38.90 ± 9.86 ^C^
CUR 50 mg/kg		4.32 ± 1.12 ^C^	2.93 ± 0.65 ^A^	2.77 ± 0.54 ^A^	2.77 ± 0.79 ^B^	143.23 ± 19.57 ^C^	43.81 ± 10.75 ^B^
CUR 100 mg/kg		3.89 ± 1.17 ^D^	3.04 ± 0.57 ^A^	2.86 ± 0.59 ^A^	3.03 ± 0.77 ^A^	141.36 ± 21.88 ^C^	49.34 ± 11.28 ^A^
						
OTC 0	CUR 0	4.08 ± 0.26	2.74 ± 0.22	2.81 ± 0.22	2.90 ± 0.21 ^b^	132.50 ± 9.65 ^e^	48.98 ± 4.61
	CUR 10	3.21 ± 0.31	3.09 ± 0.21	3.14 ± 0.26	2.96 ± 0.30 ^b^	125.82 ± 10.97 ^e,f^	51.47 ± 4.95
	CUR 50	2.83 ± 0.26	3.72 ± 0.38	3.42 ± 0.26	3.82 ± 0.37 ^a^	120.06 ± 9.07 ^e,f^	57.24 ± 6.00
	CUR 100	2.37 ± 0.24	3.72 ± 0.34	3.57 ± 0.36	4.03 ± 0.35 ^a^	114.03 ± 7.58 ^f^	63.63 ± 6.71
OTC 50	CUR 0	5.65 ± 0.49	2.00 ± 0.15	2.15 ± 0.21	1.95 ± 0.18 ^e,f^	180.63 ± 13.84 ^b^	30.52 ± 2.60
	CUR 10	5.39 ± 0.42	2.45 ± 0.20	2.40 ± 0.19	2.24 ± 0.18 ^d,e^	167.89 ± 10.04 ^b,c^	34.73 ± 3.08
	CUR 50	4.96 ± 0.42	2.68 ± 0.29	2.63 ± 0.23	2.31 ± 0.19 ^c,d,e^	150.04 ± 10.00 ^d^	39.77 ± 3.24
	CUR 100	4.36 ± 0.40	2.87 ± 0.25	2.59 ± 0.30	2.65 ± 0.24 ^b,c^	151.82 ± 9.77 ^d^	43.13 ± 2.82
OTC 75	CUR 0	6.63 ± 0.61	1.76 ± 0.20	1.69 ± 0.15	1.74 ± 0.17 ^f^	207.36 ± 11.54 ^a^	26.87 ± 1.76
	CUR 10	5.91 ± 0.69	2.04 ± 0.18	2.26 ± 0.15	2.03 ± 0.21 ^e,f^	182.04 ± 9.30 ^b^	30.52 ± 2.49
	CUR 50	5.18 ± 0.31	2.39 ± 0.24	2.27 ± 0.21	2.20 ± 0.18 ^d,e^	159.6 ± 10.32 ^c,d^	34.42 ± 3.05
	CUR 100	4.95 ± 0.37	2.53 ± 0.21	2.43 ± 0.27	2.41 ± 0.23 ^c,d^	158.24 ± 11.03 ^c,d^	41.27 ± 3.84
------------------------------------------------------------*p *values----------------------------------------------------------							
OTC		<0.001	<0.001	<0.001	<0.001	<0.001	<0.001
CUR		<0.001	<0.001	<0.001	<0.001	<0.001	<0.001
OTC × CUR		0.096	0.144	0.130	<0.001	<0.001	0.764
CUR effect for	Contrast---------------------------------------------------------------------------------------------------------						
OTC 0 mg/L	Linear	<0.001	<0.001	<0.001	<0.001	<0.001	<0.001
	Quadratic	0.022	0.063	0.303	0.458	0.914	0.280
	Cubic	0.160	0.037	0.855	0.002	0.929	0.739
OTC 50 mg/L	Linear	<0.001	<0.001	<0.001	<0.001	<0.001	<0.001
	Quadratic	0.221	0.081	0.057	0.659	0.045	0.648
	Cubic	0.966	0.536	0.448	0.091	0.122	0.551
OTC 75 mg/L	Linear	<0.001	<0.001	<0.001	<0.001	<0.001	<0.001
	Quadratic	0.139	0.289	0.002	0.546	0.001	0.088
	Cubic	0.496	0.404	0.017	0.572	0.232	0.516

MDA: Malondialdehyde (nmol/mg protein); SOD: Superoxide dismutase (U/mg protein); CAT: Catalase (k/mg protein); GSH-Px: Glutathione peroxidase (U/mg protein); GST: Glutathione S-transferase (U/mg protein); GSH: Reduced glutathione (µmol/mg protein). Data are presented as mean and standard deviation (*n* = 30 fish per treatment combination). ^x,y,z^ Differences between means indicated by different letters in the same column are statistically significant for the OTC main effect (*p* < 0.05). ^A,B,C,D^ Differences between means indicated by different capital letters in the same column are statistically significant for CUR main effect (*p* < 0.05). ^a,b,c,d,e,f^ Differences between means indicated by different letters in the same column are statistically significant for OTC × CUR interactions (*p* < 0.05).

**Table 6:** Effects of dietary curcumin supplementation against oxytetracycline-induced oxidative stress and antioxidant alterations in the spleen of rainbow trout.

Spleen		MDA	SOD	CAT	GSH-Px	GST	GSH
OTC 0 mg/L		2.47 ± 0.35 ^z^	2.89 ± 0.33 ^x^	2.91 ± 0.31 ^x^	2.78 ± 0.30 ^x^	120.08 ± 12.14 ^z^	47.49 ± 5.65 ^x^
OTC 50 mg/L		3.07 ± 0.23 ^y^	2.39 ± 0.26 ^y^	2.35 ± 0.29 ^y^	2.37 ± 0.23 ^y^	150.92 ± 15.93 ^y^	32.94 ± 4.78 ^y^
OTC 75 mg/L		3.24 ± 0.37 ^x^	2.25 ± 0.26 ^z^	2.18 ± 0.26 ^z^	2.22 ± 0.24 ^z^	167.58 ± 18.55 ^x^	27.44 ± 5.85 ^z^
						
CUR 0 mg/kg		3.21 ± 0.40 ^A^	2.28 ± 0.34 ^C^	2.22 ± 0.38 ^C^	2.28 ± 0.36 ^C^	164.74 ± 26.51 ^A^	30.96 ± 9.69 ^D^
CUR 10 mg/kg		3.00 ± 0.41 ^B^	2.45 ± 0.34 ^B^	2.42 ± 0.39 ^B^	2.44 ± 0.34 ^B,C^	147.42 ± 23.05 ^B^	33.41 ± 8.83 ^C^
CUR 50 mg/kg		2.79 ± 0.44 ^C^	2.55 ± 0.42 ^B^	2.66 ± 0.42 ^A^	2.48 ± 0.32 ^A,B^	138.88 ± 18.90 ^C^	37.45 ± 10.18 ^B^
CUR 100 mg/kg		2.71 ± 0.44 ^C^	2.75 ± 0.34 ^A^	2.62 ± 0.38 ^A^	2.62 ± 0.30 ^A^	133.74 ± 20.99 ^C^	42.00 ± 8.25 ^A^
						
OTC 0	CUR 0	2.85 ± 0.33	2.62 ± 0.32	2.66 ± 0.16	2.66 ± 0.27	132.09 ± 9.64	43.25 ± 4.29
	CUR 10	2.52 ± 0.19	2.81 ± 0.26	2.88 ± 0.29	2.79 ± 0.31	121.70 ± 8.58	44.46 ± 2.94
	CUR 50	2.33 ± 0.22	3.00 ± 0.26	3.14 ± 0.27	2.81 ± 0.32	116.59 ± 8.84	50.02 ± 5.18
	CUR 100	2.18 ± 0.22	3.11 ± 0.28	2.96 ± 0.33	2.85 ± 0.30	109.92 ± 10.15	52.24 ± 4.68
OTC 50	CUR 0	3.25 ± 0.21	2.18 ± 0.14	2.14 ± 0.24	2.21 ± 0.18	171.73 ± 9.83	27.87 ± 2.81
	CUR 10	3.16 ± 0.16	2.31 ± 0.26	2.20 ± 0.15	2.32 ± 0.18	149.65 ± 9.95	31.21 ± 2.97
	CUR 50	2.87 ± 0.19	2.46 ± 0.26	2.50 ± 0.26	2.35 ± 0.18	144.86 ± 6.77	34.57 ± 3.08
	CUR 100	2.98 ± 0.18	2.60 ± 0.18	2.55 ± 0.27	2.60 ± 0.21	137.44 ± 11.55	38.10 ± 2.91
OTC 75	CUR 0	3.54 ± 0.31	2.03 ± 0.21	1.86 ± 0.15	1.99 ± 0.21	190.39 ± 10.27	21.77 ± 1.95
	CUR 10	3.30 ± 0.30	2.23 ± 0.17	2.18 ± 0.17	2.21 ± 0.19	170.90 ± 13.62	24.56 ± 2.29
	CUR 50	3.17 ± 0.35	2.19 ± 0.21	2.33 ± 0.14	2.29 ± 0.15	155.19 ± 11.82	27.77 ± 2.88
	CUR 100	2.96 ± 0.30	2.53 ± 0.20	2.33 ± 0.22	2.41 ± 0.22	153.84 ± 9.44	35.66 ± 3.28
------------------------------------------------------------*p *values----------------------------------------------------------							
OTC		<0.001	<0.001	<0.001	<0.001	<0.001	<0.001
CUR		<0.001	<0.001	<0.001	<0.001	<0.001	<0.001
OTC × CUR		0.234	0.605	0.336	0.546	0.066	0.131
CUR effect for	Contrast---------------------------------------------------------------------------------------------------------						
OTC 0 mg/L	Linear	<0.001	<0.001	0.004	0.180	<0.001	<0.001
	Quadratic	0.259	0.634	0.024	0.633	0.531	0.717
	Cubic	0.797	0.812	0.199	0.820	0.608	0.220
OTC 50 mg/L	Linear	<0.001	<0.001	<0.001	<0.001	<0.001	<0.001
	Quadratic	0.106	0.925	0.957	0.262	0.022	0.919
	Cubic	0.030	0.951	0.143	0.233	0.154	0.970
OTC 75 mg/L	Linear	<0.001	<0.001	<0.001	<0.001	<0.001	<0.001
	Quadratic	0.890	0.259	0.006	0.363	0.016	0.004
	Cubic	0.697	0.027	0.906	0.540	0.515	0.264

MDA: Malondialdehyde (nmol/mg protein); SOD: Superoxide dismutase (U/mg protein); CAT: Catalase (k/mg protein); GSH-Px: Glutathione peroxidase (U/mg protein); GST: Glutathione S-transferase (U/mg protein); GSH: Reduced glutathione (µmol/mg protein). Data are presented as mean and standard deviation (*n* = 30 fish per treatment combination). ^x,y,z^ Differences between means indicated by different letters in the same column are statistically significant for the OTC main effect (*p* < 0.05). ^A,B,C,D^ Differences between means indicated by different capital letters in the same column are statistically significant for CUR main effect (*p* < 0.05).

**Table 7:** Effects of dietary curcumin supplementation against oxytetracycline-induced oxidative stress and antioxidant alterations in the gill of rainbow trout.

Gill		MDA	SOD	CAT	GSH-Px	GST	GSH
OTC 0 mg/L		2.52 ± 0.26 ^z^	2.78 ± 0.36 ^x^	2.03 ± 0.23 ^x^	2.63 ± 0.34 ^x^	111.30 ± 7.45 ^z^	23.91 ± 4.61 ^x^
OTC 50 mg/L		3.11 ± 0.41 ^y^	2.21 ± 0.25 ^y^	1.53 ± 0.21 ^y^	2.04 ± 0.27 ^y^	146.19 ± 17.32 ^y^	14.44 ± 2.43 ^y^
OTC 75 mg/L		3.34 ± 0.46 ^x^	2.10 ± 0.30 ^y^	1.39 ± 0.18 ^z^	1.87 ± 0.25 ^z^	157.24 ± 23.82 ^x^	12.17 ± 2.39 ^z^
						
CUR 0 mg/kg		3.37 ± 0.61 ^A^	2.16 ± 0.36 ^C^	1.47 ± 0.30 ^C^	1.92 ± 0.33 ^B^	155.55 ± 31.94 ^A^	13.20 ± 4.38 ^D^
CUR 10 mg/kg		3.05 ± 0.46 ^B^	2.26 ± 0.28 ^C^	1.59 ± 0.32 ^B^	2.04 ± 0.38 ^B^	135.53 ± 21.03 ^B^	15.42 ± 4.75 ^C^
CUR 50 mg/kg		2.80 ± 0.39 ^C^	2.43 ± 0.51 ^B^	1.75 ± 0.35 ^A^	2.32 ± 0.42 ^A^	139.09 ± 24.61 ^B^	18.60 ± 5.95 ^B^
CUR 100 mg/kg		2.73 ± 0.33 ^C^	2.60 ± 0.39 ^A^	1.79 ± 0.31 ^A^	2.44 ± 0.41 ^A^	122.79 ± 13.92 ^C^	20.13 ± 6.63 ^A^
						
OTC 0	CUR 0	2.65 ± 0.22 ^c,d,e^	2.55 ± 0.27 ^b^	1.85 ± 0.15	2.28 ± 0.21	115.71 ± 8.17 ^e,f^	18.84 ± 1.91 ^c^
	CUR 10	2.57 ± 0.29 ^c,d,e^	2.54 ± 0.15 ^b^	1.98 ± 0.21	2.51 ± 0.20	110.61 ± 5.67 ^f^	21.60 ± 1.93 ^b^
	CUR 50	2.45 ± 0.24 ^d,e^	3.04 ± 0.33 ^a^	2.16 ± 0.22	2.80 ± 0.28	112.05 ± 6.60 ^f^	26.37 ± 2.65 ^a^
	CUR 100	2.40 ± 0.22 ^e^	2.97 ± 0.35 ^a^	2.13 ± 0.20	2.92 ± 0.28	106.81 ± 7.29 ^f^	28.84 ± 3.12 ^a^
OTC 50	CUR 0	3.63 ± 0.38 ^a^	2.07 ± 0.17 ^d,e,f^	1.33 ± 0.07	1.80 ± 0.19	163.87 ± 11.71 ^b^	11.60 ± 0.95 ^f,g^
	CUR 10	3.09 ± 0.23 ^b^	2.23 ± 0.21 ^b,c,d,e^	1.41 ± 0.13	1.87 ± 0.14	153.85 ± 12.88 ^b,c^	13.32 ± 1.28 ^e,f^
	CUR 50	2.88 ± 0.28 ^b,c^	2.17 ± 0.19 ^c,d,e,f^	1.60 ± 0.19	2.19 ± 0.19	138.25 ± 10.70 ^d^	16.09 ± 1.19 ^d^
	CUR 100	2.84 ± 0.17 ^b,c,d^	2.39 ± 0.31 ^b,c,d^	1.76 ± 0.12	2.28 ± 0.19	128.77 ± 7.88 ^d,e^	16.74 ± 1.51 ^c,d^
OTC 75	CUR 0	3.84 ± 0.35 ^a^	1.84 ± 0.15 ^f^	1.21 ± 0.12	1.68 ± 0.21	187.05 ± 11.86 ^a^	9.15 ± 0.83 ^g^
	CUR 10	3.51 ± 0.24 ^a^	2.02 ± 0.21 ^e,f^	1.38 ± 0.15	1.72 ± 0.13	142.13 ± 10.77 ^c,d^	11.34 ± 1.19 ^f,g^
	CUR 50	3.08 ± 0.35 ^b^	2.08 ± 0.24 ^d,e,f^	1.49 ± 0.15	1.97 ± 0.18	166.97 ± 10.82 ^b^	13.36 ± 1.05 ^e,f^
	CUR 100	2.95 ± 0.27 ^b,c^	2.45 ± 0.20 ^b,c^	1.48 ± 0.14	2.12 ± 0.19	132.80 ± 8.63 ^d^	14.82 ± 1.13 ^d,e^
------------------------------------------------------------*p *values----------------------------------------------------------							
OTC		<0.001	<0.001	<0.001	<0.001	<0.001	<0.001
CUR		<0.001	<0.001	<0.001	<0.001	<0.001	<0.001
OTC × CUR		0.002	0.005	0.229	0.646	<0.001	<0.001
CUR effect for	Contrast---------------------------------------------------------------------------------------------------------						
OTC 0 mg/L	Linear	0.015	<0.001	<0.001	<0.001	0.015	<0.001
	Quadratic	0.802	0.783	0.214	0.492	0.976	0.857
	Cubic	0.759	0.012	0.322	0.515	0.190	0.221
OTC 50 mg/L	Linear	<0.001	0.009	<0.001	<0.001	<0.001	<0.001
	Quadratic	0.006	0.697	0.318	0.945	0.937	0.188
	Cubic	0.707	0.137	0.524	0.068	0.455	0.081
OTC 75 mg/L	Linear	<0.001	<0.001	<0.001	<0.001	<0.001	<0.001
	Quadratic	0.326	0.129	0.060	0.360	0.117	0.287
	Cubic	0.377	0.136	0.846	0.245	<0.001	0.796

MDA: Malondialdehyde (nmol/mg protein); SOD: Superoxide dismutase (U/mg protein); CAT: Catalase (k/mg protein); GSH-Px: Glutathione peroxidase (U/mg protein); GST: Glutathione S-transferase (U/mg protein); GSH: Reduced glutathione (µmol/mg protein). Data are presented as mean and standard deviation (*n* = 30 fish per treatment combination). ^x,y,z^ Differences between means indicated by different letters in the same column are statistically significant for the OTC main effect (*p* < 0.05). ^A,B,C,D^ Differences between means indicated by different capital letters in the same column are statistically significant for CUR main effect (*p* < 0.05). ^a,b,c,d,e,f,g^ Differences between means indicated by different letters in the same column are statistically significant for OTC × CUR interactions (*p* < 0.05).

## Data Availability

All data generated or analyzed during this study are included in this article, while raw data are available from the author upon request.

## References

[1] Burka J.F., Hammell K.L., Horsberg T.E., Johnson G.R., Rainnie D.J., Speare D.J. (1997). Drugs in salmonid aquaculture—A review. J. Vet. Pharmacol. Ther..

[2] Kim H.-Y., Lee I.-S., Oh J.-E. (2017). Human and veterinary pharmaceuticals in the marine environment including fish farms in Korea. Sci. Total Environ..

[3] Quesada S.P., Paschoal J.A.R., Reyes F.G.R. (2013). Considerations on the aquaculture development and on the use of veterinary drugs: Special issue for fluoroquinolones—A review. J. Food Sci..

[4] Chowdhury S., Rheman S., Debnath N., Delamare-Deboutteville J., Akhtar Z., Ghosh S., Parveen S., Islam K., Islam M.A., Rashid M.M. (2022). Antibiotics usage practices in aquaculture in Bangladesh and their associated factors. One Health.

[5] Love D.C., Fry J.P., Cabello F., Good C.M., Lunestad B.T. (2020). Veterinary drug use in United States net pen salmon aquaculture: Implications for drug use policy. Aquaculture.

[6] Leal J.F., Santos E.B.H., Esteves V.I. (2019). Oxytetracycline in intensive aquaculture: Water quality during and after its administration, environmental fate, toxicity and bacterial resistance. Rev. Aquac..

[7] Chuah L.O., Effarizah M.E., Goni A.M., Rusul G. (2016). Antibiotic application and emergence of multiple antibiotic resistance (MAR) in global catfish aquaculture. Curr. Environ. Health Rep..

[8] Yonar M.E., Yonar S.M., Silici S. (2011). Protective effect of propolis against oxidative stress and immunosuppression induced by oxytetracycline in rainbow trout (*Oncorhynchus mykiss*, W.). Fish Shellfish Immunol..

[9] Griboff J., Carrizo J.C., Bacchetta C., Rossi A., Wunderlin D.A., Cazenave J., Amé M.V. (2025). Effects of short-term dietary oxytetracycline treatment in the farmed fish *Piaractus mesopotamicus*. Environ. Toxicol. Chem..

[10] Lulijwa R., Rupia E.J., Alfaro A.C. (2020). Antibiotic use in aquaculture, policies and regulation, health and environmental risks: A review of the top 15 major producers. Rev. Aquac..

[11] El-Saadony M.T., Saad A.M., Mohammed D.M., Alkafaas S.S., Ghosh S., Negm S.H., Salem H.M., Fahmy M.A., Mosa W.F.A., Ibrahim E.H. (2025). Curcumin, an active component of turmeric: Biological activities, nutritional aspects, immunological, bioavailability, and human health benefits—A comprehensive review. Front. Immunol..

[12] Patel S.S., Acharya A., Ray R.S., Agrawal R., Raghuwanshi R., Jain P. (2020). Cellular and molecular mechanisms of curcumin in prevention and treatment of disease. Crit. Rev. Food Sci. Nutr..

[13] Kaur K., Al-Khazaleh A.K., Bhuyan D.J., Li F., Li C.G. (2024). A review of recent curcumin analogues and their antioxidant, anti-inflammatory, and anticancer activities. Antioxidants.

[14] Stohs S.J., Chen O., Ray S.D., Ji J., Bucci L.R., Preuss H.G. (2020). Highly bioavailable forms of curcumin and promising avenues for curcumin-based research and application: A review. Molecules.

[15] Komal W., Fatima S., Minahal Q., Liaqat R. (2024). Enhancing growth, antioxidant capacity, and immune response in tilapia (*Oreochromis niloticus*) through curcumin supplementation across varied stocking density paradigms. PLoS ONE.

[16] Treves-Brown K.M. (2000). Applied Fish Pharmacology.

[17] Saglam N., Ispir U., Yonar M.E. (2003). The effect of a therapeutic bath in malachite green on some haematological parameters of rainbow trout (*Oncorhynchus mykiss*, Walbaum, 1792). Fresenius Environ. Bull..

[18] Ispir U., Yonar M.E., Oz O.B. (2011). Effect of dietary vitamin E supplementation on the blood parameters of Nile tilapia (*Oreochromis niloticus*). J. Anim. Plant Sci..

[19] Siwicki A.K., Anderson D.P., Rumsey G.L. (1994). Dietary immunostimulants affect immunity in rainbow trout. Vet. Immunol. Immunopathol..

[20] Ispir U., Yonar M.E. (2007). Effects of levamisole on phagocytic activity of rainbow trout (*Oncorhynchus mykiss* W.). Acta Vet. Brno.

[21] Siwicki A.K., Anderson D.P., Siwicki A.K., Anderson D.P., Waluga J. (1993). Nonspecific defense mechanisms assay in fish: II. Potential killing activity of neutrophils and macrophages, lysozyme activity in serum and organs and total immunoglobulin level in serum. Fish Disease Diagnosis and Prevention Methods.

[22] Kajita Y., Sakai M., Atsuta S., Kobayashi M. (1990). The immunomodulatory effects of levamisole on rainbow trout, *Oncorhynchus mykiss*. Fish Pathol..

[23] Ellis A.E. (1990). Lysozyme assays. Techniques in Fish Immunology.

[24] Quade M.J., Roth J.A. (1997). A rapid, direct assay to measure degranulation of bovine neutrophil primary granules. Vet. Immunol. Immunopathol..

[25] Sahoo P.K., Kumari J., Mishra B.K. (2005). Non-specific immune responses in juveniles of Indian major carps. J. Appl. Ichthyol..

[26] Placer Z.A., Cushman L.L., Johnson B.C. (1966). Estimation of product of lipid peroxidation (malonyl dialdehyde) in biochemical systems. Anal. Biochem..

[27] Sun Y., Oberley L.W., Li Y. (1988). A simple method for clinical assay of superoxide dismutase. Clin. Chem..

[28] Aebi H. (1984). Catalase in vitro. Methods Enzymol..

[29] Beutler E. (1975). Red Cell Metabolism: A Manual of Biochemical Methods.

[30] Habig W.H., Pabst M.J., Jakoby W.B. (1974). Glutathione S-transferases: The first enzymatic step in mercapturic acid formation. J. Biol. Chem..

[31] Ellman G.L. (1959). Tissue sulfhydryl groups. Arch. Biochem. Biophys..

[32] Lowry O.H., Rosebrough N.J., Farr A.L., Randall R.J. (1951). Protein measurement with the Folin phenol reagent. J. Biol. Chem..

[33] Moradi S., Javanmardi S., Gholamzadeh P., Tavabe K.R. (2022). The ameliorative role of ascorbic acid against blood disorder, immunosuppression, and oxidative damage of oxytetracycline in rainbow trout (*Oncorhynchus mykiss*). Fish Physiol. Biochem..

[34] Abu-Zahra N.I.S., Atia A.A., Elseify M.M., Soliman S. (2024). Biological and histological changes and DNA damage in *Oreochromis niloticus* exposed to oxytetracycline: A potential amelioratory role of ascorbic acid. Aquac. Int..

[35] Kondera E., Bojarski B., Ługowska K., Kot B., Witeska M. (2020). Effects of oxytetracycline and gentamicin therapeutic doses on hematological, biochemical and hematopoietic parameters in *Cyprinus carpio* juveniles. Animals.

[36] Manna S.K., Bera A.K., Das N., Bandopadhyay C., Baitha R., Sen Ghadei S., Das B.K., Kumar A., Ravindran R., Krishna N. (2021). Determination of biosafety of the antibiotic oxytetracycline hydrochloride in *Pangasianodon hypophthalmus*. Aquac. Res..

[37] Ambili T.R., Saravanan M., Ramesh M., Abhijith D.B., Poopal R.K. (2013). Toxicological effects of the antibiotic oxytetracycline to an Indian major carp *Labeo rohita*. Arch. Environ. Contam. Toxicol..

[38] Yonar M.E., Mişe Yonar S., İspir U., Ural M.Ş. (2019). Effects of curcumin on haematological values, immunity, antioxidant status and resistance of rainbow trout (*Oncorhynchus mykiss*) against *Aeromonas salmonicida* subsp. *achromogenes*. Fish Shellfish Immunol..

[39] Giri S.S., Kim M.J., Kim S.G., Kim S.W., Kang J.W., Kwon J., Lee S.B., Jung W.J., Sukumaran V., Park S.C. (2021). Role of dietary curcumin against waterborne lead toxicity in common carp *Cyprinus carpio*. Ecotoxicol. Environ. Saf..

[40] Abdel-Tawwab M., Eissa E.-S.H., Tawfik W.A., Abd Elnabi H.E., Saadony S., Bazina W.K., Ahmed R.A. (2022). Dietary curcumin nanoparticles promoted the performance, antioxidant activity, and humoral immunity, and modulated the hepatic and intestinal histology of Nile tilapia fingerlings. Fish Physiol. Biochem..

[41] Bin-Ammar A., Ahmeda A.F., Abdelkarim M., Fath El-Bab A.F., Amer A.A., Abdelnour S.A., El-Nawsany M.M., Mahmoud A.M., Naiel M.A.E. (2024). Dietary curcumin nanoparticles improve growth performance, oxidative status and immune response of European seabass (*Dicentrarchus labrax*). Ann. Anim. Sci..

[42] Banerjee A., Kunwar A., Mishra B., Priyadarsini K.I. (2008). Concentration dependent antioxidant/pro-oxidant activity of curcumin: Studies from AAPH induced hemolysis of RBCs. Chem.-Biol. Interact..

[43] Ravikumar S., Hsieh C., Rajashekharaiah V. (2016). Prospects of curcumin as an additive in storage solutions: A study on erythrocytes. Turk. J. Med. Sci..

[44] Hossen M.S., Tanvir E.M., Prince M.B., Paul S., Saha M., Ali M.Y., Gan S.H., Khalil M.I., Karim N. (2017). Protective mechanism of turmeric (*Curcuma longa*) on carbofuran-induced hematological and hepatic toxicities in a rat model. Pharm. Biol..

[45] Rijkers G.T., Teunissen A.G., van Oosterom R., van Muiswinkel W.B. (1980). The immune system of cyprinid fish. The immunosuppressive effect of the antibiotic oxytetracycline in carp (*Cyprinus carpio* L.). Aquaculture.

[46] Guardiola F.A., Cerezuela R., Meseguer J., Esteban M.A. (2012). Modulation of the immune parameters and expression of genes of gilthead seabream (*Sparus aurata* L.) by dietary administration of oxytetracycline. Aquaculture.

[47] Abraham T.J., Julinta R.B., Roy A., Singha J., Patil P.K., Kumar K.A., Paria P., Behera B.K. (2021). Dietary therapeutic dose of oxytetracycline negatively influences the antioxidant capacity and immune-related genes expression in Nile tilapia *Oreochromis niloticus* (L.). Environ. Toxicol. Pharmacol..

[48] Kurtboğan C.Ö., Baba E., Demircan M.D. (2025). The effect of oxytetracycline on the immune response and expression of immune-related genes in European sea bass (*Dicentrarchus labrax*, L. 1758). Aquac. Res..

[49] Vargas-Chacoff L., Figueroa D., Nualart D., Muñoz J.L. (2024). The oxytetracycline and florfenicol effect on the immune system and oxidative stress response of the SHK-1 cell line of *Salmo salar*. Fishes.

[50] Mahmoud H.K., Al-Sagheer A.A., Reda F.M., Mahgoub S.A., Ayyat M.S. (2017). Dietary curcumin supplement influence on growth, immunity, antioxidant status, and resistance to *Aeromonas hydrophila* in *Oreochromis niloticus*. Aquaculture.

[51] Amer S.A., El-Araby D.A., Tartor H., Farahat M., Goda N.I.A., Farag M.F.M., Fahmy E.M., Hassan A.M., Abo El-Maati M.F., Osman A. (2022). Long-term feeding with curcumin affects the growth, antioxidant capacity, immune status, tissue histoarchitecture, immune expression of proinflammatory cytokines, and apoptosis indicators in Nile tilapia, *Oreochromis niloticus*. Antioxidants.

[52] Abdelkhalek N., El-Adl M., El-Ashram A., Othman M., Gadallah H., El-Diasty M., Dawood M.A.O., Almeer R., Abdel Daim M. (2021). Immunological and antioxidant role of curcumin in ameliorating fipronil toxicity in Nile tilapia (*Oreochromis niloticus*). Aquac. Res..

[53] Giri S.S., Sukumaran V., Park S.C. (2019). Effects of bioactive substance from turmeric on growth, skin mucosal immunity and antioxidant factors in common carp, *Cyprinus carpio*. Fish Shellfish Immunol..

[54] Ming J., Ye J., Zhang Y., Xu Q., Yang X., Shao X., Qiang J., Xu P. (2020). Optimal dietary curcumin improved growth performance, and modulated innate immunity, antioxidant capacity and related genes expression of NF-κB and Nrf2 signaling pathways in grass carp (*Ctenopharyngodon idella*) after infection with *Aeromonas hydrophila*. Fish Shellfish Immunol..

[55] Li M., Kong Y., Wu X.-M., Guo G., Sun L., Lai Y., Zhang J., Niu X., Wang G. (2022). Effects of dietary curcumin on growth performance, lipopolysaccharide-induced immune responses, oxidative stress and cell apoptosis in snakehead fish (*Channa argus*). Aquac. Rep..

[56] Li L., Shen T., Li Z., Guo Q., Pang Q. (2026). Anti-inflammatory effects of curcumin via the Nrf2-cGAS-STING-NF-κB pathway in MH7A rheumatoid arthritis fibroblast-like synoviocytes. Biomedicines.

[57] Ma F., Liu F., Ding L., You M., Yue H., Zhou Y., Hou Y. (2017). Anti-inflammatory effects of curcumin are associated with down regulating microRNA-155 in LPS-treated macrophages and mice. Pharm. Biol..

[58] Ghoushi E., Poudineh M., Parsamanesh N., Jamialahmadi T., Sahebkar A. (2024). Curcumin as a regulator of Th17 cells: Unveiling the mechanisms. Food Chem. Mol. Sci..

[59] Abdollahi E., Momtazi A.A., Johnston T.P., Sahebkar A. (2018). Therapeutic effects of curcumin in inflammatory and immune-mediated diseases: A nature-made jack-of-all-trades?. J. Cell. Physiol..

[60] Rodrigues S., Antunes S.C., Correia A.T., Nunes B. (2017). Rainbow trout (*Oncorhynchus mykiss*) pro-oxidant and genotoxic responses following acute and chronic exposure to the antibiotic oxytetracycline. Ecotoxicology.

[61] Elia A.C., Ciccotelli V., Pacini N., Dörr A.J.M., Gili M., Natali M., Gasco L., Prearo M., Abete M.C. (2014). Transferability of oxytetracycline (OTC) from feed to carp muscle and evaluation of the antibiotic effects on antioxidant systems in liver and kidney. Fish Physiol. Biochem..

[62] Xu Y.-H., Hogstrand C., Xu Y.-C., Zhao T., Zheng H., Luo Z. (2021). Environmentally relevant concentrations of oxytetracycline and copper increased liver lipid deposition through inducing oxidative stress and mitochondria dysfunction in grass carp *Ctenopharyngodon idella*. Environ. Pollut..

[63] Choudhary P., Naik S.P., Sahoo S.R., Das R., Sahoo S.N., Panda S.K., Abraham T.J., Patil P.K., Swain P., Mishra S.S. (2024). Effects of prolonged oxytetracycline supplementation on freshwater stinging catfish (*Heteropneustes fossilis*): A multi-biomarker approach. Front. Immunol..

[64] Vargas-Chacoff L., Dann F., Oyarzún-Salazar R., Nualart D., Muñoz J.L.P. (2025). Oxidative stress response of liver cell culture in Atlantic salmon challenged under two antibiotics: Oxytetracycline and florfenicol. Toxics.

[65] Vargas-Chacoff L., Ramírez-Mora J., Nualart D., Dann F., Muñoz J.L.P. (2025). Atlantic salmon (*Salmo salar*) gill primary cell culture oxidative stress and cellular damage response challenged with oxytetracycline antibiotic. Toxics.

[66] Pês T.S., Saccol E.M.H., Londero É.P., Bressan C.A., Ourique G.M., Rizzetti T.M., Prestes O.D., Zanella R., Baldisserotto B., Pavanato M.A. (2018). Protective effect of quercetin against oxidative stress induced by oxytetracycline in muscle of silver catfish. Aquaculture.

[67] Li X., Sun P., Zhang D., Yan L. (2023). Curcumin in vitro neuroprotective effects are mediated by p62/keap-1/Nrf2 and PI3K/AKT signaling pathway and autophagy inhibition. Physiol. Res..

[68] Yu X., Wu Y., Deng M., Liu Y., Wang S., He X., Allaire-Leung M., Wan J., Zou Y., Yang C. (2019). Tetracycline antibiotics as PI3K inhibitors in the Nrf2-mediated regulation of antioxidative stress in zebrafish larvae. Chemosphere.

[69] Yonar M.E. (2018). Chlorpyrifos-induced biochemical changes in *Cyprinus carpio*: Ameliorative effect of curcumin. Ecotoxicol. Environ. Saf..

[70] Bao X., Chen M., Yue Y., Liu H., Yang Y., Yu H., Yu Y., Duan N. (2022). Effects of dietary nano-curcumin supplementation on growth performance, glucose metabolism, and endoplasmic reticulum stress in juvenile largemouth bass, *Micropterus salmoides*. Front. Mar. Sci..

[71] Wu L., Dong B., Chen Q., Wang Y., Han D., Zhu X., Liu H., Zhang Z., Yang Y., Xie S. (2023). Effects of curcumin on oxidative stress and ferroptosis in acute ammonia stress-induced liver injury in gibel carp (*Carassius gibelio*). Int. J. Mol. Sci..

[72] Wu L., Zhao P., Wu P., Jiang W., Liu Y., Ren H., Jin X., Zhou X., Feng L. (2024). Curcumin attenuates ochratoxin A and hypoxia co-induced liver injury in grass carp (*Ctenopharyngodon idella*) by dual targeting endoplasmic reticulum stress and apoptosis via reducing ROS content. J. Anim. Sci. Biotechnol..

[73] Mulla A.N., Thorat S.T., Chandramore K., Kumar P., Reddy K.S., Kumar N. (2025). Curcumin as a protective agent against chromium and ammonia toxicity using molecular and biochemical approaches in fish. Sci. Rep..

[74] Li L., Zhang Z., Huang Y. (2020). Integrative transcriptome analysis and discovery of signaling pathways involved in the protective effects of curcumin against oxidative stress in tilapia hepatocytes. Aquat. Toxicol..

[75] Cui J., Li H., Zhang T., Lin F., Chen M., Zhang G., Feng Z. (2025). Research progress on the mechanism of curcumin anti-oxidative stress based on signaling pathway. Front. Pharmacol..

[76] Abrahams S., Haylett W.L., Johnson G., Carr J.A., Bardien S. (2019). Antioxidant effects of curcumin in models of neurodegeneration, aging, oxidative and nitrosative stress: A review. Neuroscience.

[77] Ashrafizadeh M., Ahmadi Z., Mohammadinejad R., Farkhondeh T., Samarghandian S. (2020). Curcumin activates the Nrf2 pathway and induces cellular protection against oxidative injury. Curr. Mol. Med..

[78] Duan C., Wang H., Jiao D., Geng Y., Wu Q., Yan H., Li C. (2022). Curcumin restrains oxidative stress of after intracerebral hemorrhage in rat by activating the Nrf2/HO-1 pathway. Front. Pharmacol..

[79] Yang S., Huang X.-L., Chen J., Mao L.-N., Liu X., Yuan W.-S., Wu X.-J., Luo G.-W. (2021). Curcumin protects BEAS-2B cells from PM2.5-induced oxidative stress and inflammation by activating NRF2/antioxidant response element pathways. Int. J. Mol. Med..

[80] Eldesoqui M., Embaby E.M., Fouad R.A., Alrajhi Y.M., Mohammed Z.A., Albadawi E.A., Al-Serwi R.H., El-Mansi A.A., Hendawy M., Ahmed M.E. (2026). Curcumin attenuates malathion-induced lung injury in rats by modulating oxidative stress, inflammation, and fibrosis via Nrf2/HO-1, NF-κB/TNF-α, and COX-2 pathways. Tissue Cell.

